# The protective effect of ginsenoside Rg1 against sepsis-induced lung injury through PI3K-Akt pathway: insights from molecular dynamics simulation and experimental validation

**DOI:** 10.1038/s41598-024-66908-y

**Published:** 2024-07-11

**Authors:** Kaiqiang Zhong, Yingui Huang, Rui Chen, Qiusha Pan, Jun Li, Xiaotu Xi

**Affiliations:** 1 Second Clinical Medical College, Guangzhou University of Chinese Medicine, Guangzhou, Guangdong China; 2 The Second Affiliated Hospital of Guangzhou University of Chinese Medicine (Guangdong Provincial Hospital of Chinese Medicine), Guangzhou, Guangdong China; 3 Guangdong Provincial Key Laboratory of Research On Emergency in TCM, Guangzhou, Guangdong China; 4 The First Affiliated Hospital of Guangzhou University of Chinese Medicine, Guangzhou, Guangdong China

**Keywords:** Ginsenoside Rg1, Sepsis, ALI, Apoptosis, Molecular dynamics simulation, Drug discovery, Pharmacology

## Abstract

Sepsis-induced acute lung injury (SALI) poses a significant threat with high incidence and mortality rates. Ginsenoside Rg1 (GRg1), derived from Ginseng in traditional Chinese medicine, has been found to reduce inflammation and protect lung epithelial cells against tissue damage. However, the specific roles and mechanisms by which GRg1 mitigates SALI have yet to be fully elucidated. In this context, we employed a relevant SALI mouse model, alongside network pharmacology, molecular docking, and molecular dynamics simulation to pinpoint GRg1's action targets, complemented by in vitro assays to explore the underlying mechanisms. Our research shows that GRg1 alleviates CLP-induced SALI, decreasing lung tissue damage and levels of serum proinflammatory factor IL-6, TNF-α, and IL-1β, also enhancing the survival rate of CLP mice. A total of 116 common targets between GRg1 and ALI, with specific core targets including AKT1, VEGFA, SRC, IGF1, ESR1, STAT3, and ALB. Further in vitro experiments assessed GRg1's intervention effects on MLE-12 cells exposed to LPS, with qRT-PCR analysis and molecular dynamics simulations confirming AKT1 as the key target with the favorable binding activity for GRg1. Western blot results indicated that GRg1 increased the Bcl-2/Bax protein expression ratio to reduce apoptosis and decreased the high expression of cleaved caspase-3 in LPS-induced MLE-12 cells. More results showed significant increases in the phosphorylation of PI3K and AKT1. Flow cytometric analysis using PI and Annexin-V assays further verified that GRg1 decreased the apoptosis rate in LPS-stimulated MLE-12 cells (from 14.85 to 6.54%, *p* < 0.05). The employment of the AKT1 inhibitor LY294002 confirmed these trends, indicating that AKT1’s inhibition negates GRg1’s protective effects on LPS-stimulated MLE-12 cells. In conclusion, our research highlights GRg1's potential as an effective adjunct therapy for SALI, primarily by inhibiting apoptosis in alveolar epithelial cells and reducing pro-inflammatory cytokine secretion, thus significantly enhancing the survival rates of CLP mice. These beneficial effects are mediated through targeting AKT1 and activating the PI3K-AKT pathway.

## Introduction

Sepsis is a life-threatening condition caused by an uncontrolled and dysregulated systemic immune response to microbial infections that poses a significant global health burden. The mortality rate for sepsis among hospitalized patients is alarmingly high, ranging from 30 to 45%^1^. Acute lung injury (ALI), characterized by impaired gas exchange due to pneumonia and tissue damage, is one of the most common and severe manifestations that contributes to the increased sepsis morbidity and mortality rate^2^. The development of sepsis-induced ALI (SALI) often involves the apoptosis of alveolar epithelial cells (AECs) and the disruption of the alveolar-capillary barrier^3^. In cases of viral sepsis, especially those caused by COVID-19, the virus primarily targets the alveolar epithelial cells. The consequent cell apoptosis contributes to an uncontrolled inflammatory response, leading to the build-up of protein-laden edematous fluid within the lungs. Such accumulation aggravates pulmonary edema and can escalate into acute respiratory distress syndrome (ARDS)^4^. The current therapeutic approaches for SALI are primarily restricted to anti-infection measures and respiratory support^5^. Targeting the apoptosis of alveolar epithelial cells may emerge as a promising method to alleviate the severity of SALI^6^.

Ginseng (Panax ginseng C. A. Mey.), a cornerstone in traditional Chinese medicine (TCM), is leveraged extensively for the management of sepsis. Formulations like the Shen-Fu injection, which incorporate ginseng as a key constituent, are officially recognized by the National Administration of TCM and are routinely employed in the clinical setting for the treatment of septic shock^7^. Ginsenoside Rg1 (GRg1), a principal bioactive compound found in ginseng, is noted for its capacity to modulate a variety of biological targets while conferring minimal adverse effects^8^. The extant literature corroborates the anti-inflammatory, anti-apoptotic, and immunomodulatory properties of GRg1^9^. Earlier studies have reported that GRg1 is documented as significantly reducing the levels of pro-inflammatory cytokines, including TNF-α, IL-6, iNOS, and IL-1β, while simultaneously enhancing the synthesis of the anti-inflammatory cytokine IL-10^10^. In mouse models, the administration of GRg1 has been observed to suppress the release of pro-inflammatory cytokines and mitigate lung tissue damage^11,12^. Although several studies have elucidated the protective role of GRg1 in SALI, the detailed molecular underpinnings of its action continue to be poorly understood.

**Figure 1:**
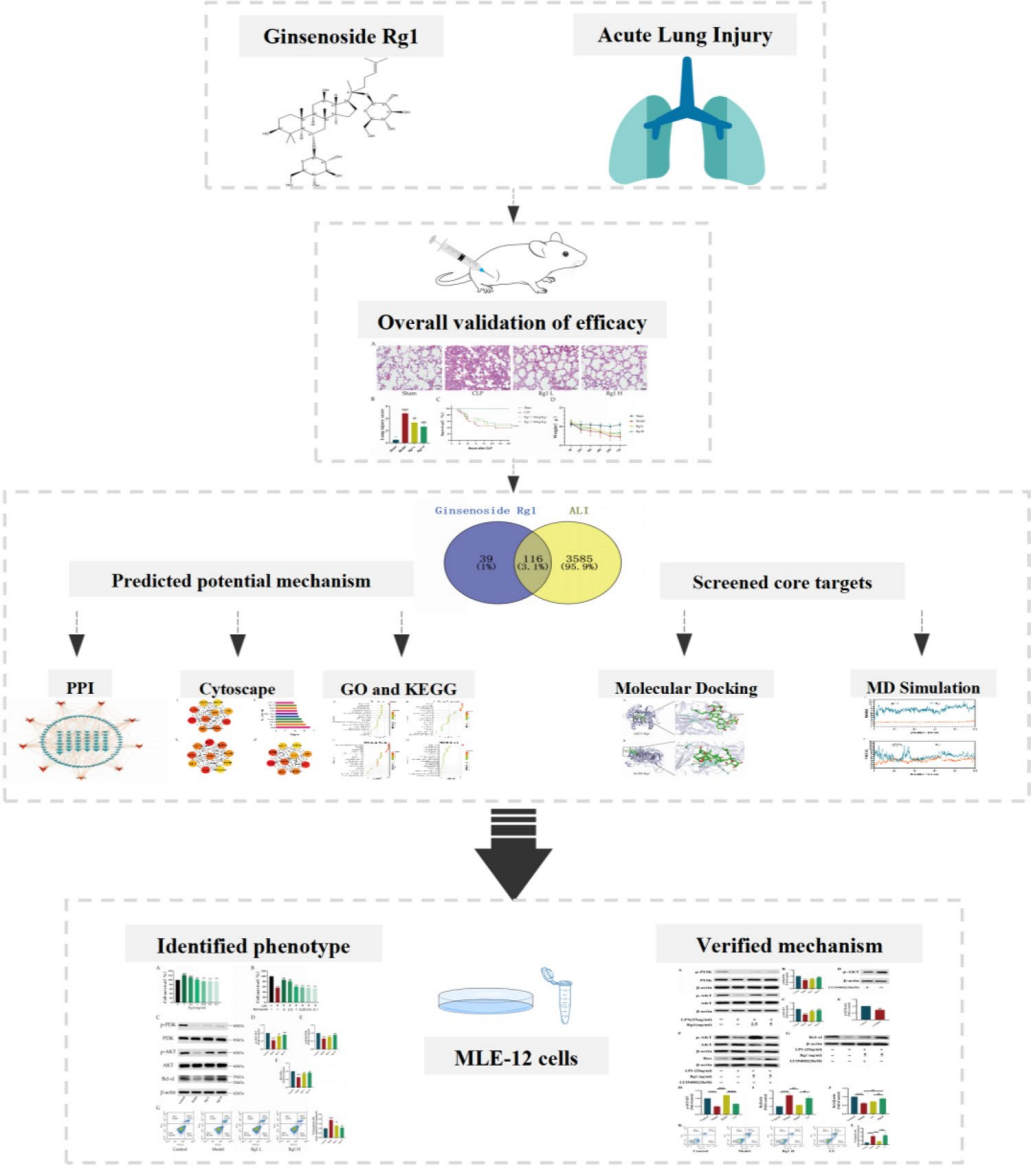
Flow chart showing the experimental design.

Therefore, this study aims to elucidate the anti-inflammatory effects of GRg1 and its mechanisms in preventing lung injury in SALI. Using animal models and MLE-12 cells to assess the regulatory role of GRg1 on SALI. Additionally, network pharmacology and molecular docking are employed to identify potential drug action targets, thereby clarifying the underlying mechanisms of GRg1's effects (Fig. 1).

## Results

### GRg1 attenuates acute lung injury resulting from cecal ligation and puncture (CLP) in vivo

#### GRg1 improve survival rate in CLP-induced ALI mice model

**Figure 2:**
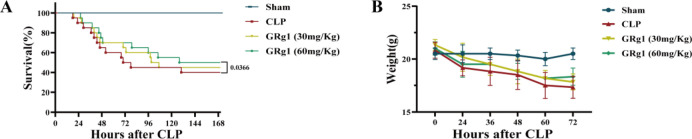
GRg1 could attenuate the lung injury induced by CLP. (**A**,** B**) The 7-day survival and 3-day weight changes of each group of mice were monitored. ***p  < 0.001 vs. sham, ****p  < 0.0001 vs. sham, ##*p *< 0.01 vs. CLP, ###*p * < 0.001 vs. CLP and ####*p * 0.0001 vs. CLP.

We implemented the cecal ligation and puncture (CLP) procedure to induce polymicrobial sepsis in a murine model. Subsequently, the mice were stratified into four distinct cohorts: the sham controls; the CLP group; the CLP plus GRg1 treatment at a dose of 30 mg/Kg (Rg1 30 mg/Kg group); and the CLP plus GRg1 treatment at a higher dose of 60 mg/Kg (Rg1 60 mg/Kg group). Mice that received GRg1 underwent bi-daily intraperitoneal administration of their respective doses for a total of seven days. The variance in survival rates between the groups receiving lower and higher doses of GRg1 did not reach statistical significance (*p* > 0.05). But, mice that received the higher dose of GRg1 exhibited a substantial decrease in seven-day mortality rates (*p* < 0.05), along with amelioration of weight loss within the initial 72 h following the CLP procedure (*p* < 0.05), as illustrated in Fig. 2A,B.

#### GRg1 relieves pulmonary pathological damage and reduces apoptosis in CLP-induced injury

**Figure 3:**
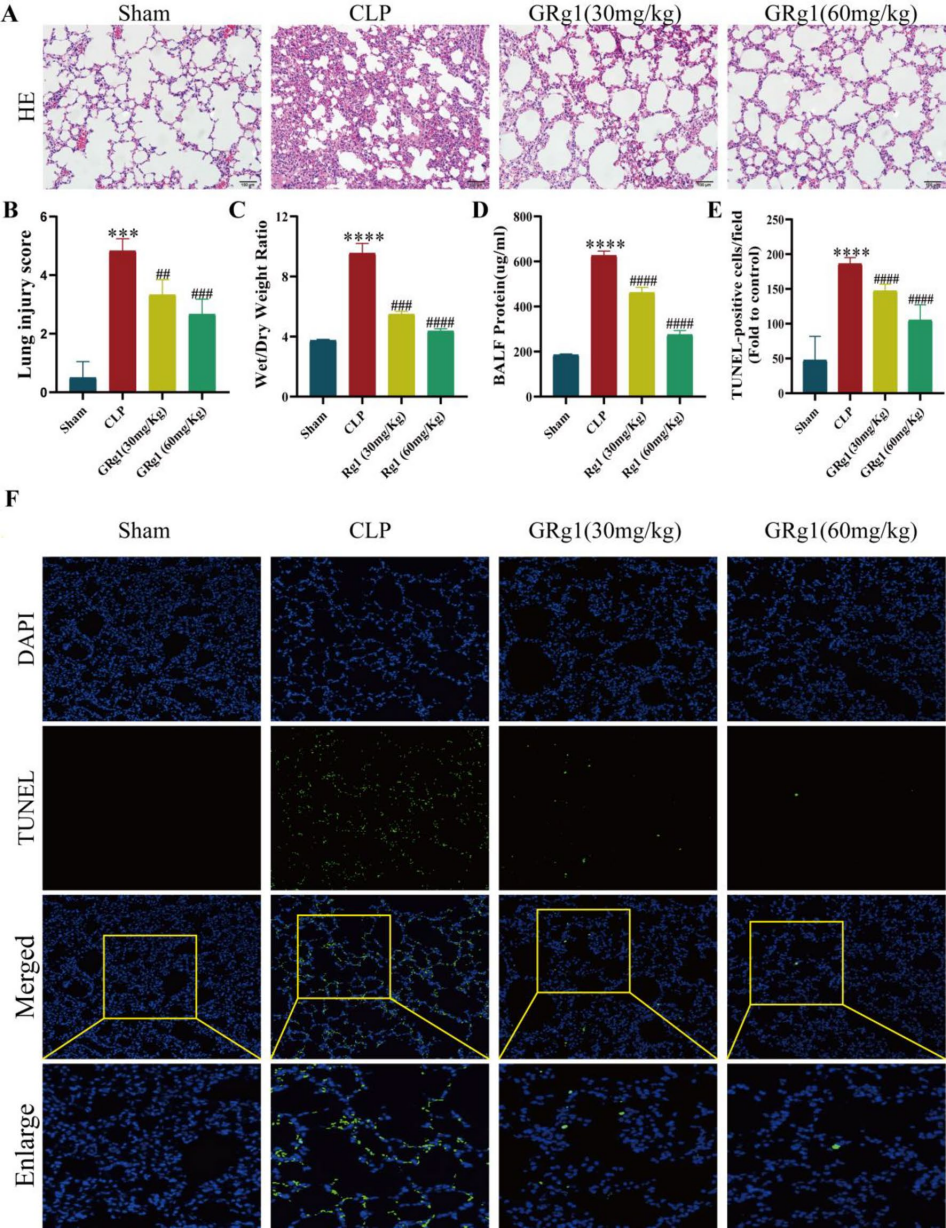
GRg1 could attenuate the lung injury induced by CLP. (**A**) H&E staining and TUNEL staining were used to assess GRg1's effect on lung histology. Scale magnification × 400 (n = 6); (**B**) Lung injury scores were measured in the lungs; (**C**) Lung wet/dry (W/D) ratio. Dissected lung tissues were weighed and oven dried at 80 ℃ for 48 h for calculation of W/D ratio. (D) The total protein concentration in supernatant was measured by the BCA method. (E–F) Apoptosis in CLP mice effectively alleviated with the treatment of GRg1. ***p  < 0.001 vs. sham, ****p  < 0.0001 vs. sham, ## *p * 0.01 vs. CLP, ###*p * < 0.001 vs. CLP and #### *p * 0.0001 vs. CLP.

To further investigate the therapeutic effects of GRg1, mice with ALI were treated with GRg1, and lung tissue was collected 48 h post-surgery and stained with H&E. As expected, compared to the sham-operated group, LPS stimulation induced significant histological changes, including interstitial edema, thickening of the alveolar walls, and extensive infiltration of inflammatory cells in the alveolar spaces. However, GRg1 treatment significantly alleviated these CLP-induced pathological changes (*p* < 0.05) (Fig. 3A–D).

To further evaluate the protective effect of GRg1 in experimental ALI, we quantitatively analyzed several parameters related to CLP-induced pulmonary edema and microvascular permeability. These parameters include the lung W/D weight ratio and protein concentration in BALF. GRg1 treatment essentially eliminated CLP-induced pulmonary edema and microvascular permeability, as evidenced by the significant reduction in the lung W/D weight ratio (*p* < 0.05) (Fig. 3C) and protein content in BALF (*p* < 0.05) (Fig. 3D). These results indicate that GRg1 strongly protects alveolar-vascular barrier integrity, reduces pulmonary edema, and mitigates LPS-induced cellular damage. Furthermore, TUNEL staining revealed a significant number of apoptotic cells in the lung tissue of CLP-induced ALI, whereas GRg1 effectively reduced the number of apoptotic cells (*p* < 0.05) (Fig. 3E,F).

#### GRg1 attenuates pro-inflammatory factors in vivo

**Figure 4:**
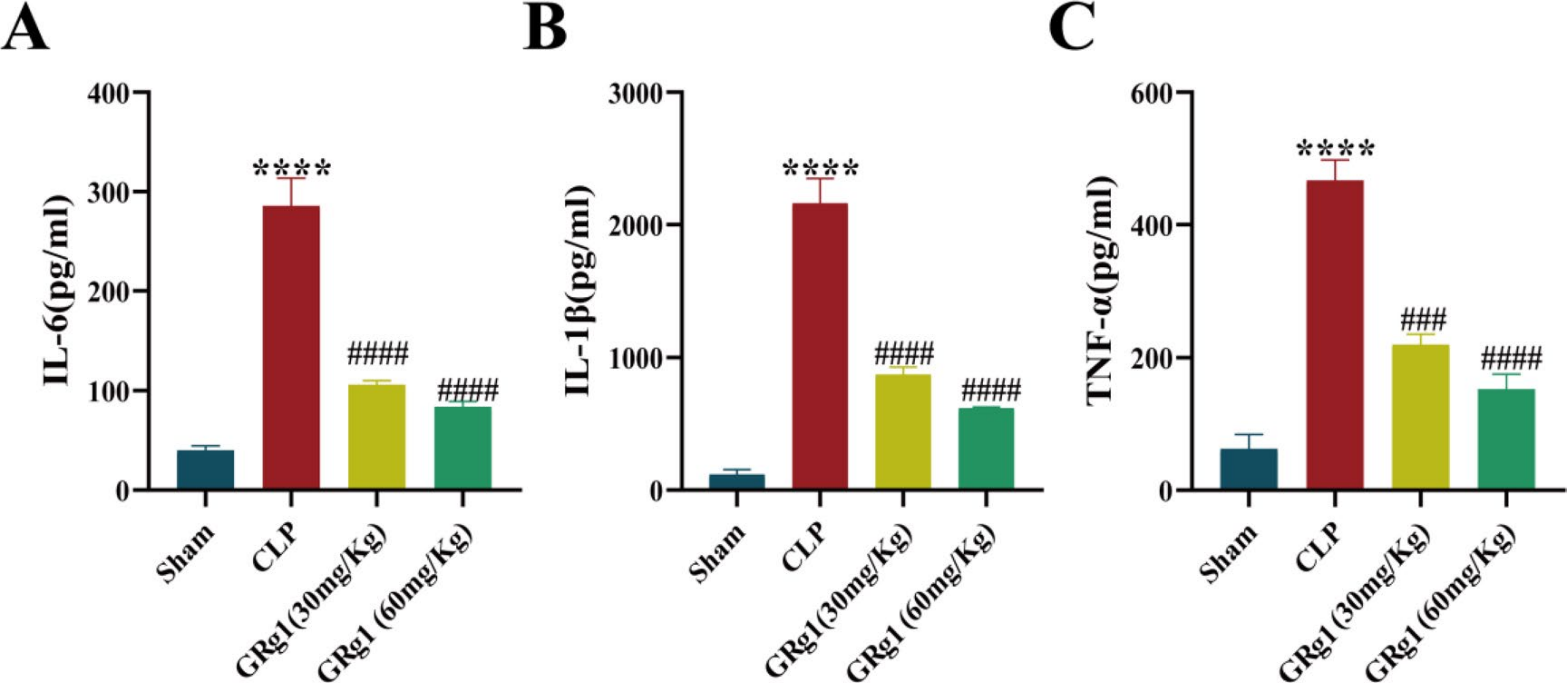
GRg1 could attenuate the lung injury induced by CLP. (**A**–**C**) Influence of GRg1 on the concentrations of IL-6, TNF-α, and IL-1β in serum of mice with CLP-induced acute lung injury. ***p < 0.001 vs. sham, ****p < 0.0001 vs. sham, ##*p* < 0.01 vs. CLP, ###*p * < 0.001 vs. CLP and ####*p * < 0.0001 vs. CLP.

The levels of pro-inflammatory cytokines, including IL-6, TNF-α, and IL-1β, in the serum were measured in mice 48 h post-CLP procedure (Fig. 4A–C). The results highlighted a significant surge in the production of the aforementioned inflammatory cytokines in the CLP group compared to the sham group. Notably, the GRg1 treatment, at both dosage levels, was correlated with a graded decrease in the serum concentrations of these pro-inflammatory mediators when contrasted with the model group (*p* < 0.05).

### AKT1 as a key target for GRG1 to ameliorate SALI damage by Network Analysis

#### PPI network analysis and central target screening

**Figure 5:**
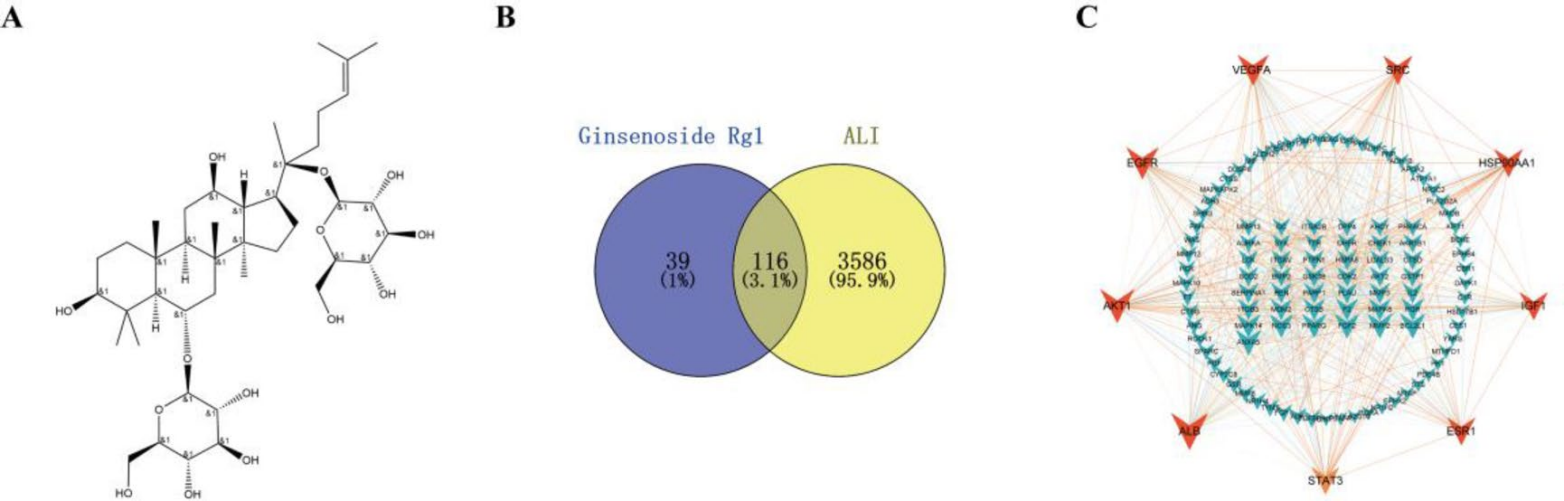
The structure of GRg1 and identification of shared targets of GRg1 in combating ALI. (**A**) Two-dimensional 2D structure of GRg1; (**B**) Potential targets of GRg1 against ALI; (**C**) The PPI network is visualized by Cytoscape.

The two-dimensional structures of GRg1 are illustrated in Fig. 5A, and their pharmacological and molecular properties were obtained from the TCMSP database (Supplementary Materials Table S1). GRg1 has a molecular weight of 801.14 and a drug-likeness (DL) score of 0.28. A drug-likeness weight of ≥ − 0.18 generally indicates favorable pharmacokinetic properties^13^. Our compilation of 155 targets associated with GRg1 was obtained through SwissTargetPrediction (with a probability threshold of > 0) and PharmMapper (with a Norm Fit of > 0.5). In the exploration of targets linked to ALI, 3702 targets were identified using the Genecards, OMIM, and TDD databases. Furthermore, we found 116 common targets (Fig. 5B and Supplementary Table S2) between GRg1 and ALI.

Using the STRING tool, a comprehensive analysis was performed. The constructed network comprised 113 nodes and 910 edges, exhibiting an average node degree of 16.1, and was depicted as a protein–protein interaction (PPI) network through the use of Cytoscape software (Fig. 5C). To further refine the results, the core targets were identified using the CytoHubba extension (Supplementary Materials Figure S1). By overlapping the targets identified in the four screening results, several central targets were detected, including AKT1, EGFR, VEGFA, STAT3, IGF1, ESR1, ALB, SRC, and HSP90AA1, among others. The prominent target genes identified in this research have crucial roles in the development and potential management of ALI, highlighting their significance in mediating the therapeutic effects of GRg1.

#### GO and KEGG enrichment analysis

**Figure 6:**
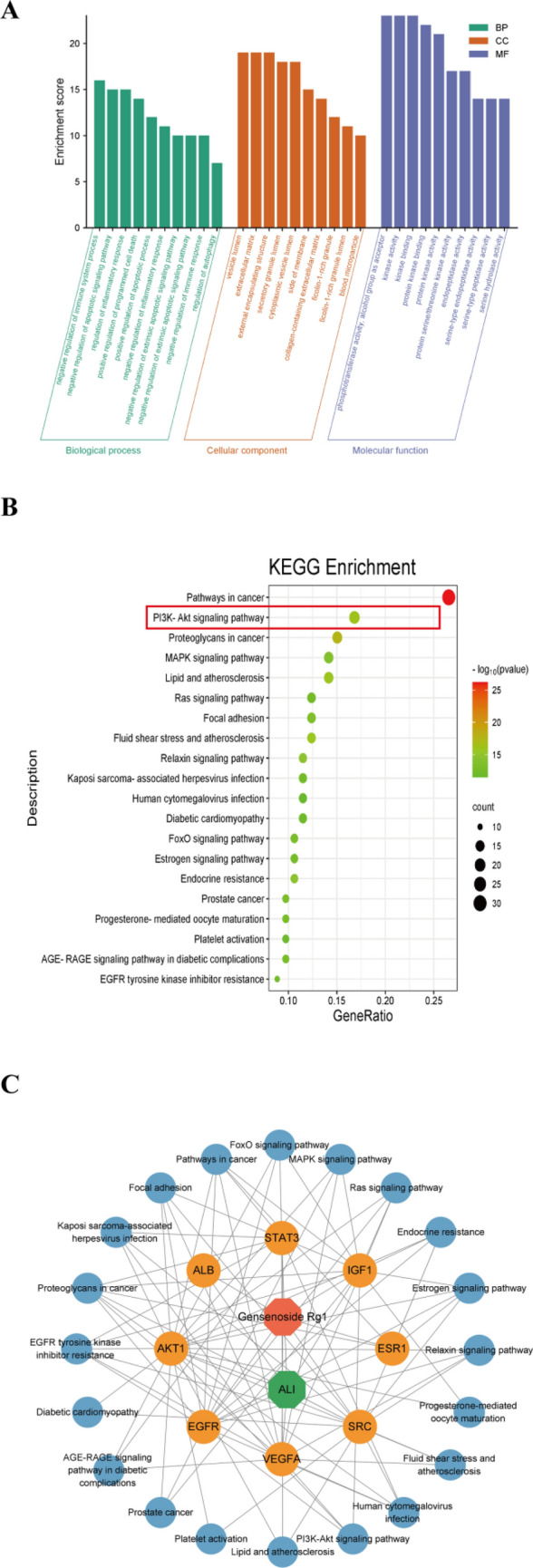
Enrichment analysis of GRg1 against ALI using GO and KEGG. (**A**)The top 10 terms for biological processes (BP), cellular components (CC), and molecular functions (MF) in the GO enrichment analysis are represented by green, orange, and purple bars, respectively; (**B**) The bubble chart displays the top 20 pathways identified through KEGG enrichment analysis; (**C**) Component-Disease-Target-Pathway Interaction Network. Red represents GRg1, green represents ALI, orange represents the core targets, and blue represents the pathways.

A total of 116 target genes were analyzed using Metascape, leading to the identification of the top 20 enriched terms through Gene Ontology (GO) analysis (Fig. 6A). Simultaneously, we conducted a KEGG pathway enrichment analysis, unveiling the top 20 potential signaling cascades highlighted in Fig. 6B. Foremost among the up-regulated biological processes (BPs) identified were those pertinent to the negative regulation of the immune system process, and the attenuated regulation of the apoptotic signaling pathway, among others. Regarding cellular components (CCs), the notable up-regulation involved entities such as vesicle lumen, extracellular matrix, and cytoplasmic vesicle lumen, as well as various membrane segments. On the molecular function (MF) front, an upsurge in activities was noted in protein kinase binding, kinase activity specific to proteins, and protein serine/threonine kinase activity. Within the realm of the KEGG pathways, significant enrichment was observed in pathways such as PI3K-Akt, MAPK, and Ras, which represent potential key pathways for treating SALI using GRg1.

Finally, we constructed a component-Disease-Target-Pathway Interaction Network to visualize that GRg1 potentially influences ALI by modulating multiple targets and signaling pathways (Fig. 6C), including AKT1, EGFR, VEGFA, STAT3, IGF1, ESR1, ALB, SRC, the PI3K-Akt signaling pathway, the MAPK signaling pathway, and the Ras signaling pathway.

### Molecular docking analysis between GRg1 and the key shared targets

**Figure 7:**
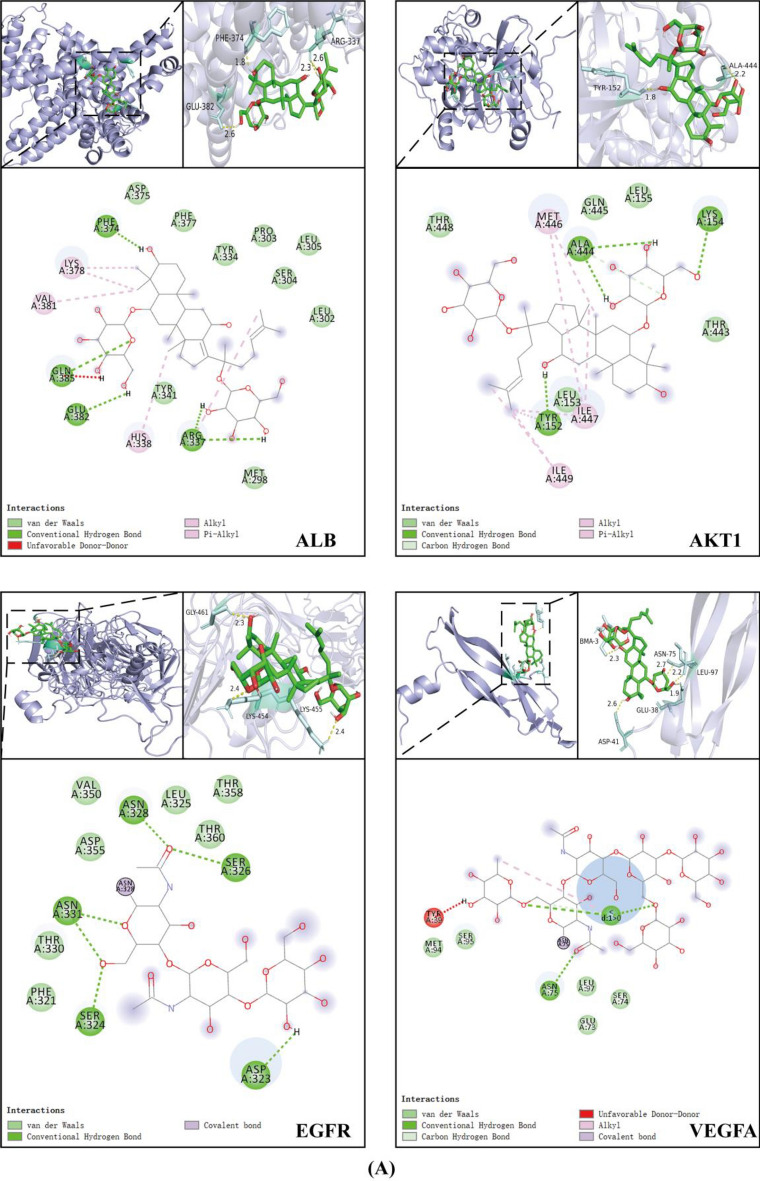

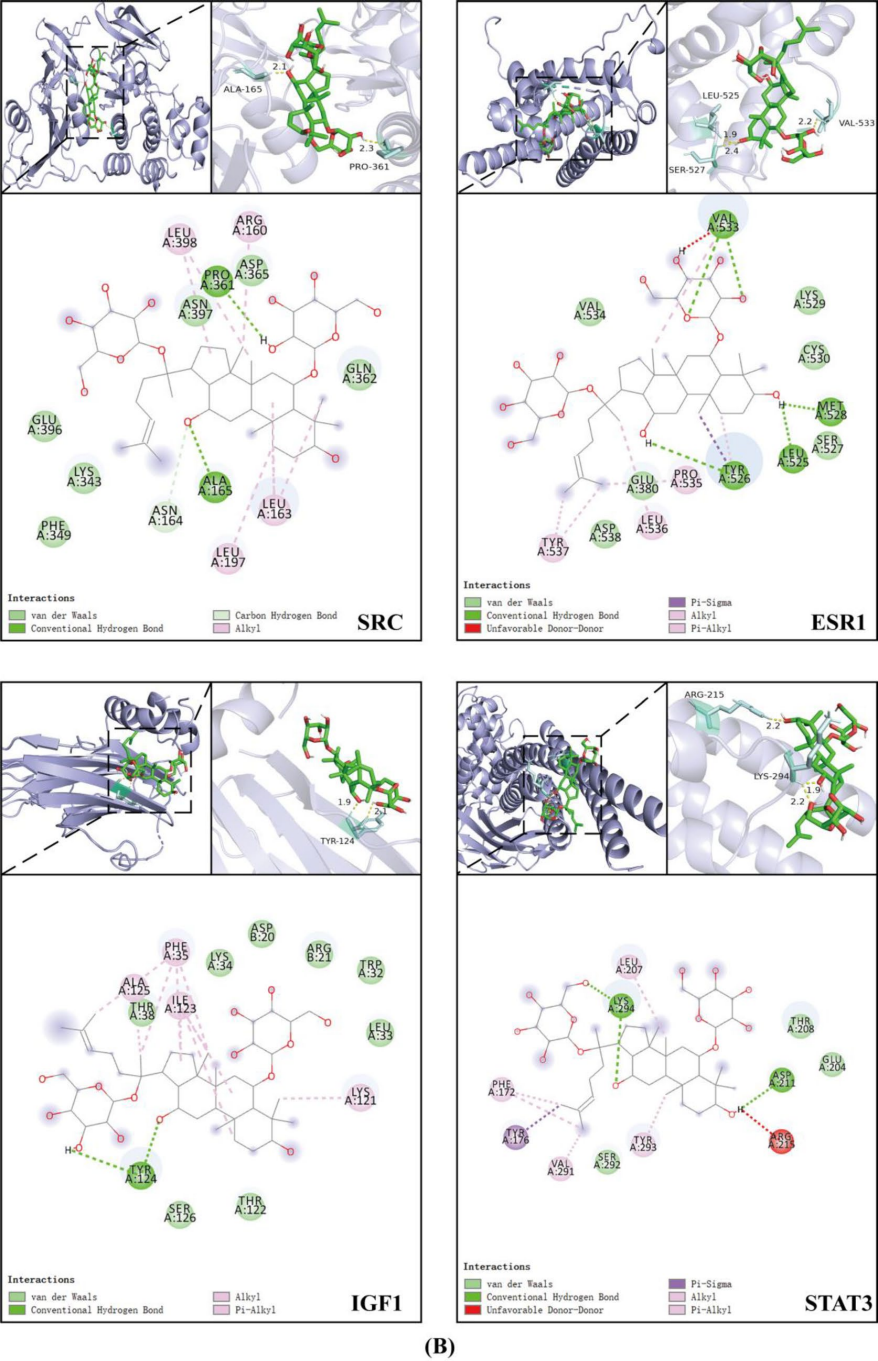

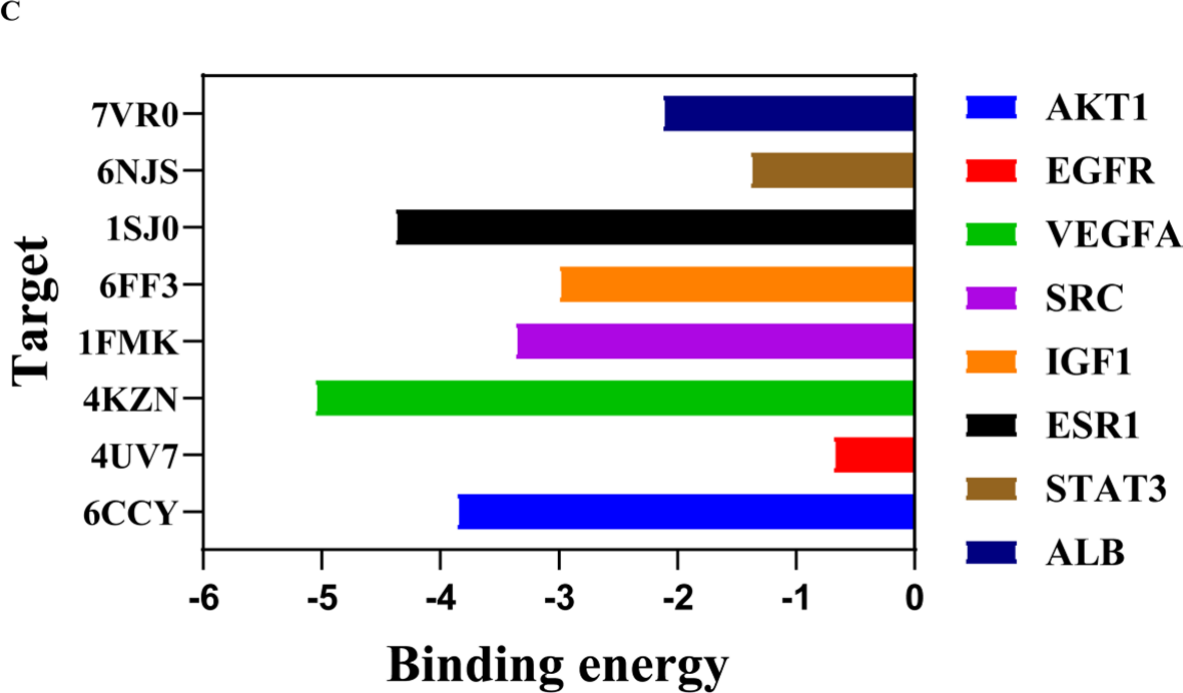
The complex conformations of GRg1 with eight proteins: (**A**) ALB, AKT1, EGFR, and VEGFA. (**B**) SRC, ESR1, IGF1, and STAT3; (**C**) Molecular docking binding energies. Each protein complex display consists of three parts: 1. The three-dimensional conformation of GRg1 with each protein complex, with GRg1 depicted in green and the protein in blue; 2. Residues around GR1 involved in conventional hydrogen bonds are shown. Different atoms are labeled with distinct colors. The carbon (C) of GRg1 and the protein are colored purple and pink, respectively. Hydrogen (H), sulfur (S), oxygen (O), and nitrogen (N) atoms are represented in white, yellow, red, and blue, respectively.

Molecular docking was performed to verify the binding activity of GRg1 with core targets, including AKT1, EGFR, VEGFA, SRC, IGF1, ESR1, STAT3, and ALB, using AutoDockTools 1.5.6 (Fig. 7C; Table S3). To delve into the complex binding interactions, visualizations were created using both PyMOL and Discovery Studio 2019, as depicted in Fig. 7A,B. Our analysis indicated that the preponderance of the targeted proteins, encompassing AKT1, VEGFA, SRC, IGF1, ESR1, STAT3, and ALB, exhibited binding energies surpassing the benchmark of stability set at − 1.2 kcal/mol, except for EGFR. The outcome showed a stable conformational structure and strong binding affinity between GRg1 and these proteins. These findings further supported the potential of GRg1 as an active constituent capable of effectively interacting with the identified key targets.

### Screening of potential targets for Grg1 effect on MLE-12 cells

#### GRg1-related targets in MLE-12 cells after LPS stimulation identified qRT-PCR

To evaluate the therapeutic efficacy of GRg1 on the aforementioned targets in SALI, we established a cell-based assay using the murine lung epithelial cell line, MLE-12, which was stimulated with lipopolysaccharides (LPS). After LPS induction, the cells were treated with GRg1. We assessed the mRNA expression levels of AKT1, VEGFA, SRC, IGF1, ESR1, STAT3, and ALB through quantitative real-time PCR (qRT-PCR) analysis.

**Figure 8:**
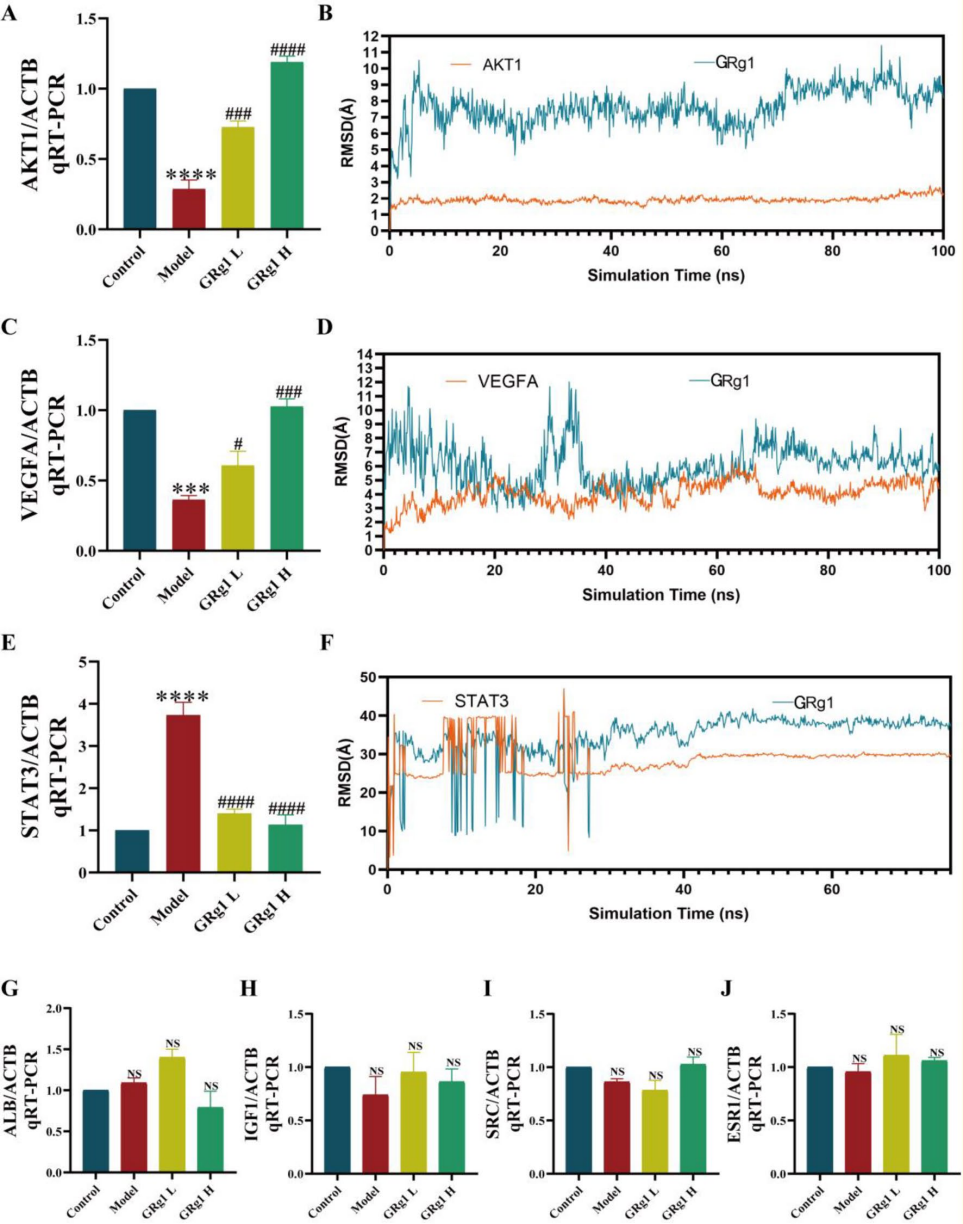
Analysis of GRg1 Key Target Expression in MLE-12 Cells via RT-qPCR and RMSD correlation with Critical Targets. (**A**) AKT1; (**B**) (6CCY)-GRg1; (**C**) VEGFA; (**D**) VEGFA (4KZN)-GRg1; (**E**) STAT3. (**F**) STAT3 (6NJS)-GRg1; (**G**) ALB; (**H**) IGF1; (**I**) SRC; (**J**) ESR1. ***p < 0.001 vs. Control, ****p < 0.0001 vs. Control, #*p* < 0.05 vs. Model, ##*p* < 0.01 vs. Model, ###p < 0.001 vs. Model and ####*p* < 0.0001 vs. Model. Control (untreated, n = 3); Model (LPS-treated, n = 3); GRg1L (GRg1L treated cell at 2.5 ug/ml, n = 3); GRg1L (GRg1L treated cell at 5 ug/ml, n = 3).

Our study revealed that LPS stimulation resulted in an up-regulation of STAT3 expression (as depicted in Fig. 8E), alongside a down-regulation of VEGFA (Fig. 8A) and AKT1 (Fig. 8C) expression in MLE-12 cells, with these changes reaching statistical significance (*p* < 0.05). In contrast, the GRg1 treatment mitigated the effects induced by LPS, as it significantly decreased STAT3 levels while enhancing the expression of AKT1 and VEGFA (*p* < 0.05). There were no notable differences in the expression levels of ALB, IGF1, SRC, and ESR1 among the four study groups (Fig. 8G–J). These findings underscore the potential of STAT3, AKT1, and VEGFA as prospective therapeutic targets for GRg1 in treating SALI.

#### A potential interaction between GRg1 and AKT1 by Molecular dynamics simulation

The stability of the protein–ligand complexes was investigated using molecular dynamics simulations. The study involved 100 ns molecular dynamics simulations for the AKT1 (6CCY)-Rg1, STAT3 (6NJS)-Rg1, and VEGFA (4KZN)-Rg1 complexes, aiming to assess the dynamics, trajectories, structural characteristics, binding affinities, and conformational changes in the molecules based on the docking results and their validation. The root-mean-square deviation (RMSD) serves as a dependable measure for evaluating the stability of protein–ligand complexes, quantifying the extent to which atomic positions vary from their original configurations. A lower RMSD value indicates enhanced conformational stability^14^. The RMSD alterations in the complexes were analyzed. The RMSD of the AKT1 protein remained consistently within the range of 1.8–2.4 Å (Fig. 8B), and the RMSD of the STAT3 protein remained relatively stable at approximately 28 Å (Fig. 8F); meanwhile, the RMSD fluctuation range of the VEGFA protein was between 7.5 and 10.5 Å (Fig. 8D). AKT1 and GRg1 achieved a more stable binding conformation through molecular dynamics simulation, leading to increased affinity and stability in this specific conformation. Thus, AKT1 is a fundamental element of the PI3K-Akt signaling pathway, which is thought to be intrinsically linked to the apoptosis of alveolar epithelial cells.

### GRg1 reduces alveolar epithelial cell apoptosis by engaging the PI3K-Akt signaling pathway in vitro

#### GRg1 mitigate apoptosis in MLE-12 cells prompted by LPS exposure

**Figure 9:**
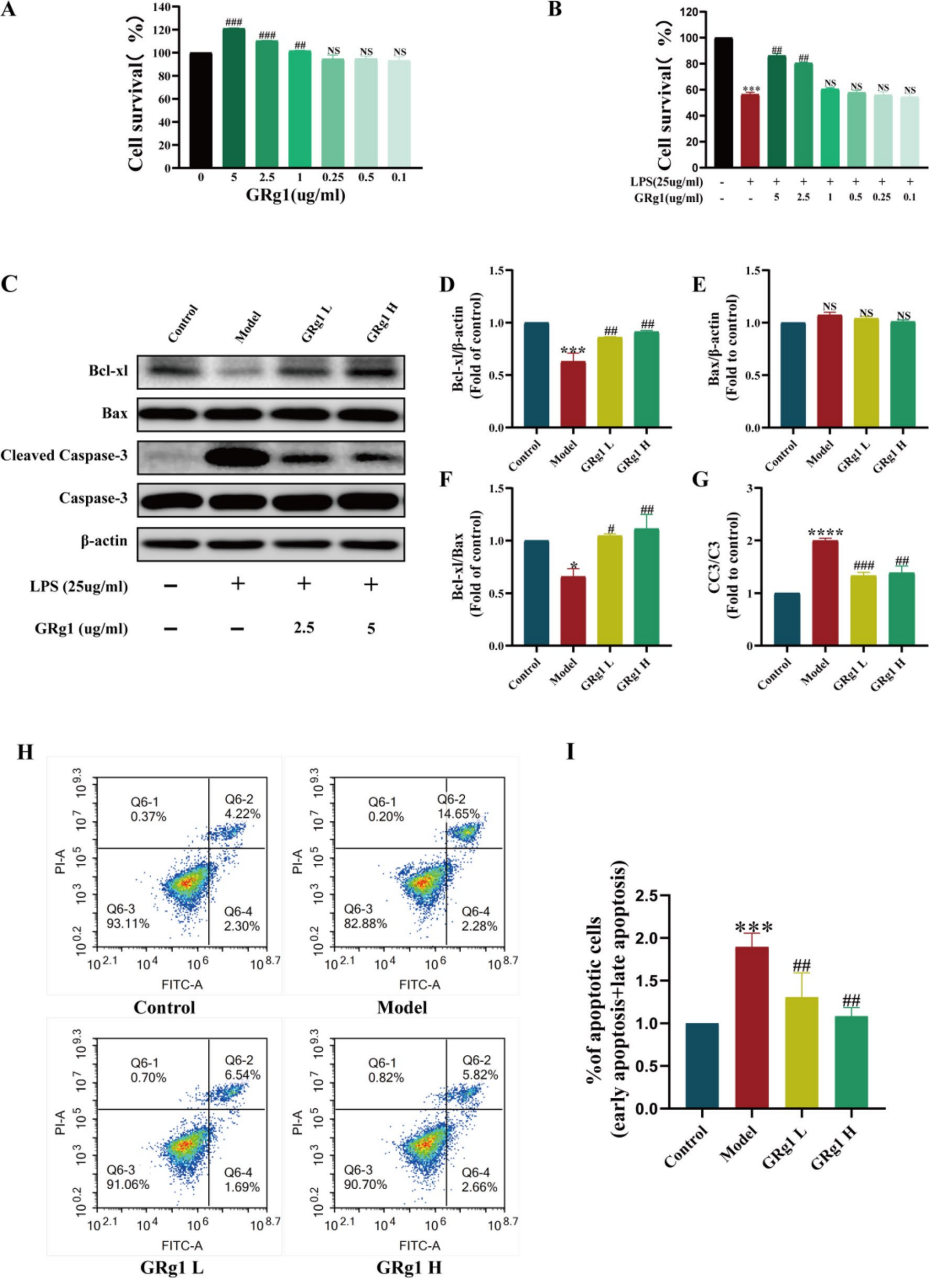
GRg1 Attenuation of LPS-Induced Apoptosis in MLE-12 Cells. (**A**) CCK-8 analysis was used to detect the viability of MLE-12 cells exposed to different concentrations of GRg1 for 24 h. ##p < 0.01, ###p < 0.001 vs 0 mg/ml (n = 6); (**B**) The viability of MLE-12 cells stimulated with GRg1 and LPS for 24 h was determined via CCK-8 analysis. ***p < 0.001 vs control, ##*p* < 0.01 vs LPS group (n = 6); (**C**) Bcl-xl, caspase-3, cleaved-caspase-3, and Bax expression in MLE-12 cell groups analyzed by Western blot analysis (n = 3), the samples derive from the same experiment and that blots were processed in parallel; (**D**) The expression of Bcl-xl; (**E**) The expression of Bax; (**F**) Bcl-xl/Bax ratio; (**G**) cleaved-caspase-3/ caspase-3 ratio; (**H**,** I**) Flow Cytometric Assessment of Early and Late Apoptosis in LPS-Treated MLE-12 Cells. *p < 0.05 vs. Control, ***p < 0.001 vs. Control, ****p < 0.0001 vs. Control, #*p* < 0.05 vs. Model, ##*p* < 0.01 vs. Model and ###*p* < 0.001 vs. Model.

Cell apoptosis is implicated in the pathogenesis of SALI^15^. Within this framework, results in vivo suggested that GRg1 could mitigate pulmonary pathology and apoptosis in models of CLP-induced injury. Therefore, we investigated the anti-apoptotic effects of GRg1 on MLE-12 cells. Initially, a cytotoxicity assessment was performed using different GRg1 concentrations (0, 0.1, 0.25, 0.5, 1, 2.5, and 5 µg/ml) (Fig. 9A). The findings indicated negligible cytotoxicity, with concentrations of 2.5 and 5 µg/ml GRg1 showing a notable promotion of cell proliferation (*p* < 0.05). Following this, an LPS-induced model of cellular stress was generated utilizing a concentration of 25 µg/ml of LPS. Treatment with GRg1, at both 5 µg/ml and 2.5 µg/ml, resulted in a significant reduction in LPS-mediated cellular activity inhibition (*p* < 0.05, Fig. 9B). Consequently, the investigation proceeded with 2.5 µg/ml of GRg1 for the low-dose group and 5 µg/ml of GRg1 for the high-dose group.

Further experiments involved subjecting MLE-12 cells to 25 µg/ml LPS for 24 h. Subsequent analysis centered on the ratio between the anti-apoptotic protein, Bcl-xl, and the pro-apoptotic protein, Bax, recognized as an apoptosis marker^16^. This was evaluated via Western blotting (WB). In parallel, caspase-3 activation, an apoptotic hallmark, was appraised. GRg1 treatment was found to significantly reverse both the LPS-induced decline in the Bcl-xl/Bax ratio and the elevation in cleaved caspase-3 levels (*p* < 0.05, Fig. 9C–G).

In the final phase, apoptosis was quantified through flow cytometry by analyzing apoptosis rates in the Q2 regions via the PI and Annexin-V assay (Fig. 9H–I). The data reflected a substantive increase in apoptosis levels due to LPS (*p* < 0.05), which GRg1 effectively ameliorated (*p* < 0.05). Apoptotic cell proportions were 4.22%, 14.85%, 6.54%, and 5.82% for the control, model, LPS + Rg1 low-dose (2.5 µg/mL), and LPS + Rg1 high-dose (5 µg/mL) groups, respectively.

#### GRg1 effects in LPS-stimulated MLE-12 cells through the PI3K-Akt signaling pathway

The KEGG enrichment analysis results indicated that the PI3K-Akt signaling pathway was the most significant, apart from the cancer-related pathways. Molecular docking and dynamic simulations further corroborated a robust affinity and stable interaction between GRg1 and AKT1. Consequently, our investigation honed in on the Akt signaling cascade to discern the anti-apoptotic influence of GRg1 in the context of LPS-challenged MLE-12 cells.

**Figure 10:**
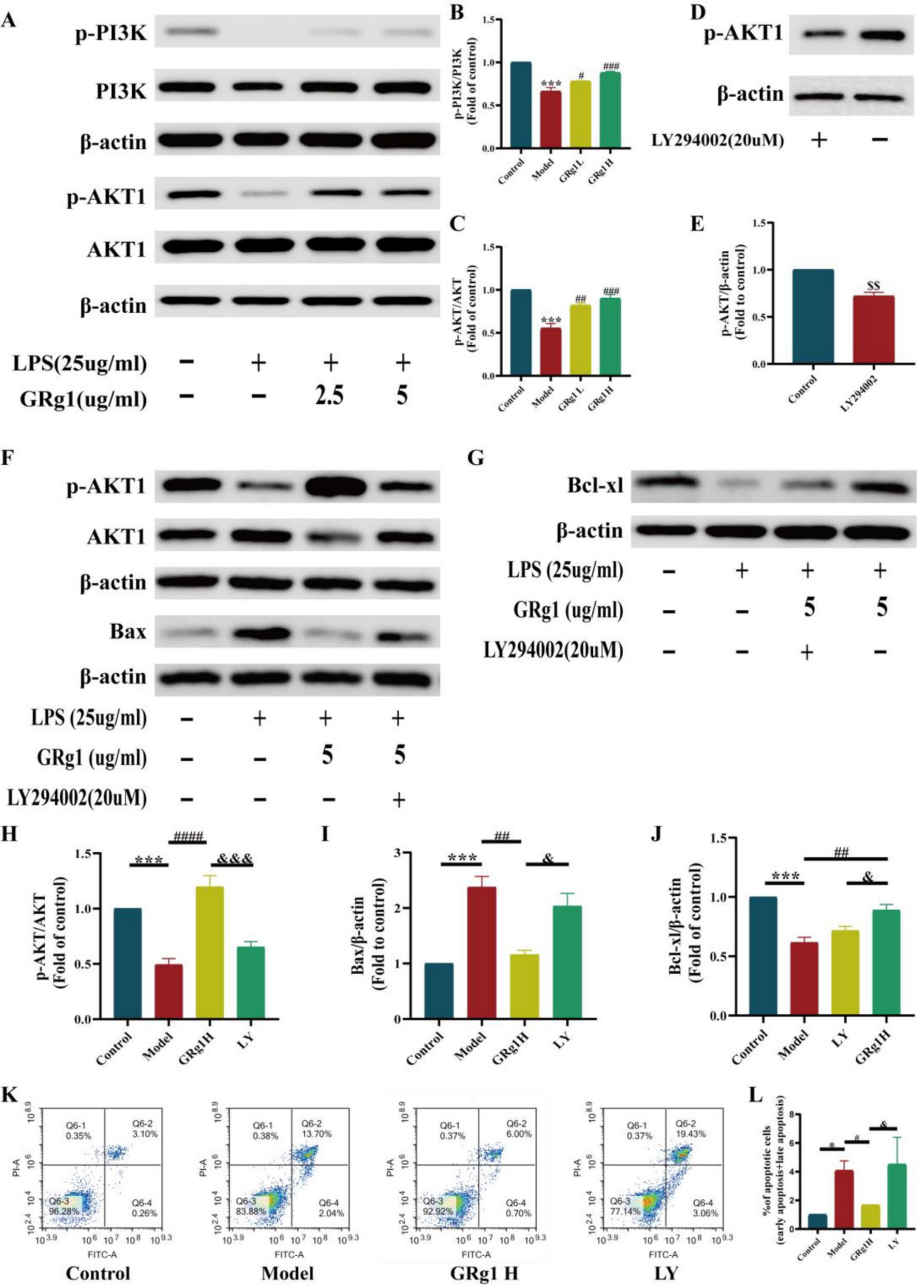
GRg1 exerts an anti-apoptotic function in MLE-12 cells via the Akt signaling pathway. (**A**–**C**) The level of phosphorylated Akt and phosphorylated PI3K proteins were assessed through immunoblotting and quantitatively analyzed in MLE-12 cells treated with LPS (25ug/ml) and GRg1 (2.5ug/ml and 5ug/ml); (**D**, **E**) Western blotting was employed to assess the expression of p-Akt in MLE-12 cells upon stimulation with LY294002 (20uM). &&p < 0.01 vs. control; (**F**–**J**) Western blotting was utilized to detect the protein expression of LPS (25ug/ml), GRg1 H (5ug/ml), and LY294002 (20uM) in stimulated MLE-12 cells, followed by quantitative analysis; (**K**, **L**) The impact of LPS (25ug/ml), GRg1 H (5ug/ml), and LY294002 (20uM) on the apoptosis rate of MLE-12 cells was evaluated using flow cytometry. Statistical analysis was performed, and the following significance levels were observed. *p < 0.05 vs. Control, ***p < 0.001 vs. Control, #*p *< 0.05 vs. Model, ##*p* < 0.01 vs. Model, ###*p* < 0.001 vs. Model and ####p < 0.0001 vs. Model; &* p*< 0.05 vs. GRg1 H; &&&*p* < 0.001 vs. GRg1 H. The samples were derived from the same experiment and the blots were processed in parallel.

Commencing the analysis, we quantified the protein levels of phosphorylated PI3K (p-PI3K) and PI3K, and phosphorylated AKT1 (p-AKT1) and AKT1 post-treatment with either LPS or LPS alongside GRg1. Western blot analysis demonstrated that LPS exposure notably diminished the phosphorylation of PI3K and AKT1 compared to the control group, which was significantly restored by the addition of GRg1 (*p* < 0.05, Fig. 10A–C). These results endorse the notion that GRg1 may activate the PI3K-Akt signaling pathway within MLE-12 cells.

Subsequent explorations focused on whether the anti-apoptotic effect of GRg1 on LPS-induced ALI was mediated via the PI3K-Akt pathway. To this end, MLE-12 cells underwent treatment with the specific Akt inhibitor LY294002 before Western blotting, which confirmed the successful suppression of AKT1 phosphorylation by LY294002 (*p* < 0.05, Fig. 10D–E). Thereafter, MLE-12 was subjected to treatment combinations of LPS, GRg1, and LY294002. Western blotting indicated that GRg1 countered the LPS-induced reduction in Bcl-xl levels and Bcl-xl/Bax ratio and the upregulation of Bax, which was then annulled by LY294002 concurrent with the inactivation of AKT1 (Fig. 10F–J). Consistent with these findings, flow cytometric analysis corroborated the exacerbation of apoptosis rates in MLE-12 cells stimulated with LPS and GRg1 when LY294002 was present (*p* < 0.05, Fig. 10K–L).

Collectively, these observations illuminate the biological processes and molecular interactions potentially modulated by GRg1, offering substantive insights into its prospective therapeutic modality in the context of SALI management.

#### The role of GRg1 in CLP induced ALI through the PI3K/AKT signaling pathway

**Figure 11:**
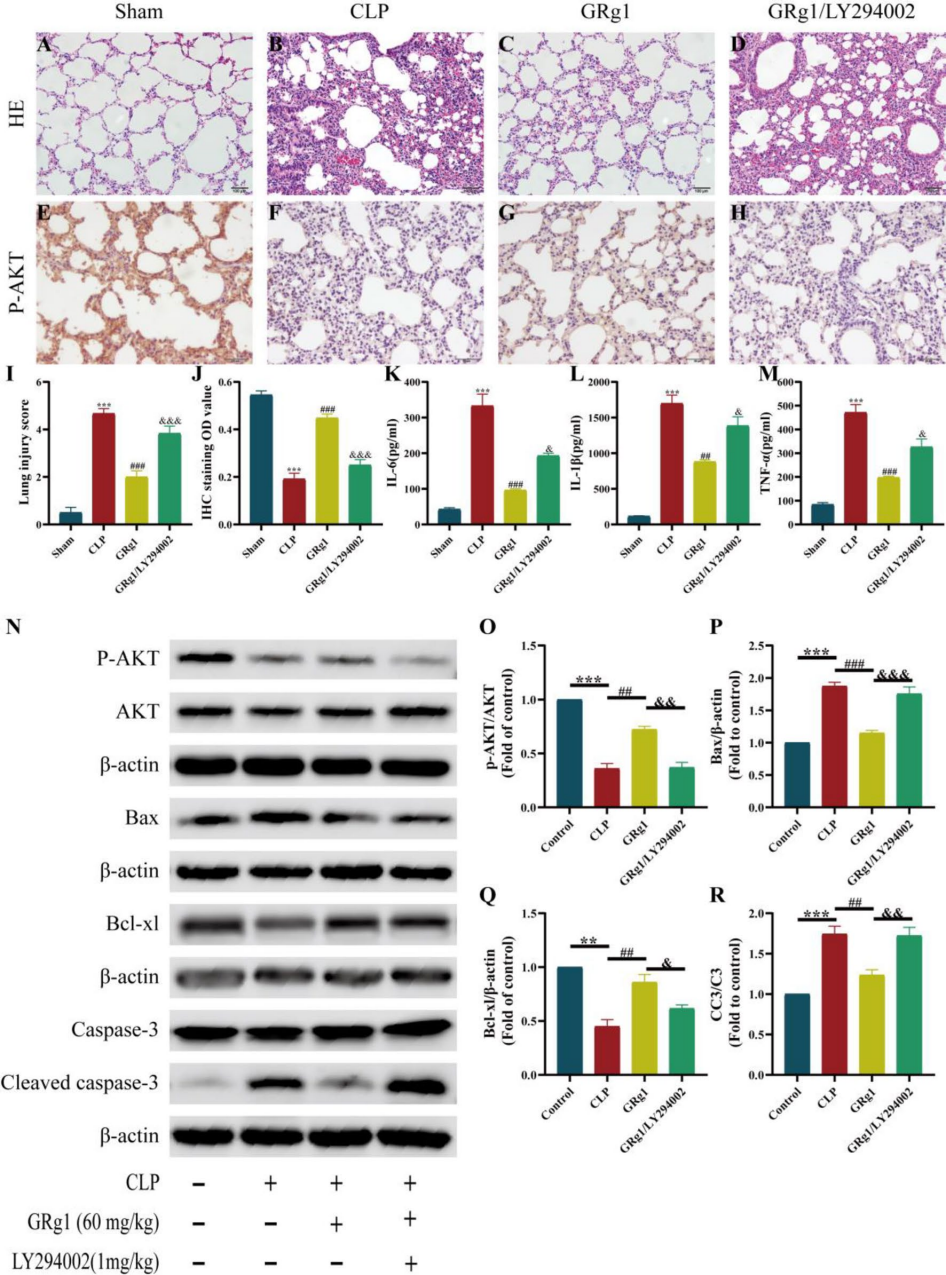
GRg1 attenuates CLP-induced ALI by PI3K/Akt pathway in vivo. (**A**–**D**) Lung tissue sections were stained with H&E for histopathologic analysis (magnification 400 × , n = 6); (**E**–**H**) Immunohistochemistry staining of p-Akt protein (magnification 400 × , n = 8). (**I**) lung injury score; (**J**) Representative images of p-Akt protein level in lung determined by immunohistochemistry; (**K**–**M**) GRg1 significantly reduces the production of pro-inflammatory cytokines IL-6, IL-1β, and TNF-α in serum through the PI3K/AKT pathway; (**N**) Western blotting was used to assess the levels of p-AKT,AKT,Bax,Bcl-xl,caspase-3 and cleaved caspase-3; (**O**–**R**) Densitometric quantification of the level of p-AKT/AKT,Bax,Bcl-xl and CC3/C3. Statistical analysis was performed, and the following significance levels were observed. *p < 0.05 vs. Control, ***p < 0.001 vs. Control, #*p* < 0.05 vs. Model, ##*p* < 0.01 vs. Model and ###*p* < 0.001 vs. Model; &*p* < 0.05 vs. GRg1; &&*p* < 0.01 vs. GRg1 and &&&*p* < 0.001 vs. GRg1. The samples were derived from the same experiment and the blots were processed in parallel.

To verify whether the protective effect of GRg1 on ALI is reversed when the PI3K/AKT signaling pathway is inhibited, we first examined the histopathological and immunohistochemical staining of the inhibitor LY294002. As expected, compared to the Con group, CLP significantly increased the number of inflammatory cells in the alveolar cavity, while GRg1 treatment significantly alleviated these CLP-induced pathological changes. However, after administering the inhibitor LY294002, there was a substantial infiltration of inflammatory cells in the lung tissue, with noticeable edema in the lung interstitium (Fig. 11A–D,I). Immunohistochemical staining showed that GRg1 promotes the expression of *p*-AKT, while LY294002 partially inhibits the expression of p-AKT (Fig. 11E–H,J). ELISA results for inflammatory factors IL-6, IL-1β, and TNF-α in serum indicated that LY294002 can reverse the inhibitory effect of GRg1 on these inflammatory factors(Fig. 11K–M). Further, WB analysis of lung tissue showed that, compared to the Con group, CLP significantly reduced the p-AKT/AKT ratio and the expression of the anti-apoptotic protein Bcl-xl, while increasing the ratio of apoptotic proteins Bax and CC3/C3. GRg1 treatment altered these results, but the combined use of the inhibitor LY294002 reversed the therapeutic effects of GRg1(Fig. 11N–R). These findings indicate that the LY294002 inhibitor can interfere with the anti-ALI effect of GRg1 pretreatment, suggesting that this inhibitor may block the PI3K/ AKT signaling pathway, thus interfering with its protective effect on lung tissue. Therefore, PI3K/ AKT is likely the primary signaling pathway regulated by GRg1.

## Discussion

The persistent inflammation characteristics of sepsis harbor the potential to escalate into acute lung injury (ALI), precipitating irreversible pulmonary damage. The association of sepsis-induced ALI with increased mortality rates in comparison to ALI from other etiologies is of particular note. ALI, and its more severe form, acute respiratory distress syndrome (ARDS), presents significant hindrances to survival, incited by various innate and extraneous factors. The current understanding is that the pathology encompasses widespread damage to the pulmonary interstitium and alveolar epithelial cells, with mortality reaching as high as 40% in affected individuals^17^. In this light, obstructing the progression of ALI is vital to ameliorating the severity of sepsis. Within the detrimental milieu of septic ALI, the process of apoptosis plays a noteworthy role^18^. Emerging studies have suggested that curbing apoptosis in pulmonary epithelial cells may offer a viable strategy to alleviate lung inflammation and injury^19,20^. Ginsenoside Rg1 has surfaced in recent pharmacological discourse as a compound proficient in blunting inflammatory and apoptotic cascades^12,21,22^. However, its explicit function and the mechanisms by which it influences sepsis-associated ALI require further elucidation. Thus, our study sought to clarify the impacts and possible mechanisms of GRg1 on this condition.

Our in vivo experiments, GRg1 was administered to septic mice, resulting in significantly reduced mortality and weight loss. Histopathological H&E staining, lung tissue W/D ratio, protein content in bronchoalveolar lavage fluid, and TUNEL staining provided evidence that GRg1 significantly reduced tissue damage and apoptosis. ELISA assays confirmed GRg1's ability to inhibit pro-inflammatory mediators, including IL-6, TNF-α, and IL-1β. These observations support the hypothesis that GRg1’s protective effect against SALI may operate through mechanisms that prevent apoptosis, thereby regulating the secretion of inflammatory factors.

Our comprehensive approach, combining network pharmacology and molecular docking, delved into the complex mechanisms of GRg1 in ALI. Analysis of relevant databases revealed potential key targets and pathways associated with ALI, highlighting the regulation of immune responses, apoptosis, and inflammation. Proteins involved in these processes were further scrutinized, and PPI network analysis identified eight central targets. Subsequent molecular docking, in vitro validation, and dynamic simulation confirmed that AKT1 is the most stable binding target of GRg1. KEGG pathway enrichment emphasized the critical role of the PI3K-Akt pathway in cell survival.

Our in vitro results demonstrated that GRg1 could reverse LPS-induced apoptosis in mouse lung epithelial cells (MLE-12), normalize the Bcl2/Bax ratio, and increase levels of cleaved caspase-3 post-LPS administration. Western blot analysis confirmed that GRg1 mitigates LPS-induced apoptosis in MLE-12 cells by modulating the PI3K-Akt pathway. To verify the crucial role of AKT1, we used the AKT-specific inhibitor LY294002, which blocked the AKT pathway and negated the anti-apoptotic effects of GRg1 on MLE-12 cells. This supports the notion that GRg1 alleviates lung injury via the PI3K-Akt pathway by inhibiting apoptosis.

Further in vivo investigation into the effects of GRg1 on the PI3K/AKT pathway revealed that the anti-inflammatory and anti-apoptotic effects of GRg1 were reversed when the PI3K/AKT signaling pathway inhibitor LY294002 was administered. Therefore, these results demonstrate that GRg1 has a protective effect against sepsis-induced acute lung injury, mediated through the activation of the PI3K/AKT pathway.

## Materials and methods

### Animals

Female Balb/c mice, aged 6 to 8 weeks and weighing between 20 to 25 g, were obtained from Vital River Laboratories, China. All experimental mice were housed at the Experimental Animal Center of Guangdong Provincial Hospital of Chinese Medicine (SYXK 2018–0094, Guangdong). The mice were kept in a controlled environment with a temperature maintained at 22 ± 1 °C, relative humidity at 50 ± 1%, and a 12-h light/12-h dark cycle. All animal handling procedures, including the euthanasia protocol, followed the standards and protocols of the Institutional Animal Care and Use Committee of Guangdong Provincial Hospital of Chinese Medicine and were in accordance with the principles set by AAALAC and IACUC. The animal research protocol was approved by the Animal Care and Use Committee of Guangdong Provincial Hospital of Chinese Medicine (Guangzhou, China) under permit number 2020071. Animal welfare and experimental protocols were conducted in accordance with the ARRIVE guidelines and strictly adhered to the Guide for the Care and Use of Laboratory Animals of the United States.

### Establishment of the SALI mouse model

The procedure of cecal ligation and puncture (CLP) was adopted as a model for simulating polymicrobial sepsis, with the methodology in this study aligned with previously described processes^23^. BALB/c mice were divided into four groups: Sham group, CLP group, and two CLP groups receiving either a high dose or low dose of GRg1 (n = 20/group). Before surgery, animals were fasted for at least 12 h and then anesthetized with inhaled isoflurane. In the SALI and GRg1 intervention groups, a midline laparotomy was performed to expose the cecum, which was then punctured twice with an 18-gauge needle and ligated below the ileocecal valve. The Sham group underwent the same surgical exposure without CLP. Postoperative subcutaneous saline infusion was provided to all cohorts. The designated treatment groups received daily intraperitoneal injections of GRg1 at 30 mg/kg for the low-dose group or 60 mg/kg for the high-dose group, starting 7 days before the CLP intervention^21,24,25^. The control group underwent CLP alone and received an equivalent volume of saline.

Seven days post-CLP, survival rates and body weight changes were recorded. Additionally, lung tissue samples and serum were extracted for further analysis two hours after the second day's treatment. During the observation period following successful modeling, we assessed whether the mice exhibited intolerable pain or significant declines in mobility, which were defined as humane endpoints. Mice meeting these criteria were euthanized by inhalation of excess isoflurane, and the experiment was immediately terminated. At the end of the experiment, all mice were anesthetized with excess isoflurane and then euthanized.

For signaling pathway elucidation assays, we used an inhibitor to block the PI3K/Akt signaling pathway. Male Balb/c mice were randomly divided into the following four groups (n = 8 per group): (1) Control group; (2) CLP group; (3) CLP + GRg1 (60 mg/kg/d; i.p.) group; (4) CLP + GRg1 + LY294002 group. Mice in the CLP + GRg1 group received intraperitoneal injections of GRg1 for 7 days prior to CLP modeling. The CLP + GRg1 + LY294002 group was pretreated with the PI3K/Akt pathway inhibitor LY294002 (1 mg/kg) one hour before CLP modeling. The inhibitor dose was predetermined in preliminary experiments and had no significant impact on lung function.

### Histological studies

The histopathological examination of lung tissues was conducted on the mice. The right upper lobe of the lung was collected and fixed in 4% paraformaldehyde for at least 24 h. Paraffin-embedded Sects. (4-5 μm) were prepared and stained with hematoxylin and eosin after deparaffinization and rehydration. The slides were blindly scored by experienced pathologists under an optical microscope using the following criteria: 0-normal tissue; 1-mild inflammation; 2-no significant damage to lung structure; 3-thickening of alveolar septa; 4-formation of nodules or areas of distorted normal structure; and 5-complete loss of the field of view^26^.

### TUNEL staining

The pulmonary tissues were preserved in 4% paraformaldehyde solution and then encased within paraffin blocks. TUNEL assay was performed using a one-step TUNEL apoptosis detection kit (Beyotime, Jiangsu, China) to detect apoptotic cells through TUNEL staining, and DAPI was used to stain the cell nucleus. Images were captured using a fully automated inverted fluorescence microscopy system (ECLIPSE Ti2-E; NIKON, Japan).

### Immunohistochemical analysis

The presence of phosphorylated protein kinase B (p-Akt) in paraffin-embedded sections was detected by immunohistochemical staining^27^. Hydrated paraffin sections were incubated in a blocking solution (10% normal rabbit serum + 5% skim milk + 3% BSA + 0.1% Triton X-100) for 10 min, then incubated with anti-p-AKT (#4060) overnight at 4 °C. After rinsing with PBS (pH 7.4), the sections were incubated with horseradish peroxidase (HRP)-conjugated goat anti-rabbit IgG antibody (1:200) at room temperature for 50 min. The sections were then incubated with diaminobenzidine on a chromogenic substrate, counterstained with hematoxylin, and observed using an Olympus IX73 microscope system (Tokyo, Japan). The extent of immunopositive staining was evaluated using ImageJ.

### Serum levels of IL-6, TNF-α, and IL-1β were measured using enzyme-linked immunosorbent assay (ELISA)

Blood serum was extracted, and the quantities of inflammatory cytokines present in the serum of the mice were evaluated using an ELISA kit (CUSABIO, China).

### Predicting potential gene targets of ginsenoside Rg1

The chemical structure (canonical SMILES) and 2D structure of ginsenoside Rg1 were downloaded from PubChem (https://pubchem.ncbi.nlm.nih.gov/). Subsequently, the chemical structure was imported into SwissTargetPrediction (http://www.swisstargetprediction.ch/) and PharmMapper (http://www.lilab-ecust.cn/pharmmapper/) to identify drug compound targets. To ensure accuracy, any duplicated targets were removed from the analysis.

### ALI-associated target genes

To screen disease gene targets associated with ALI, the keyword "acute lung injury" was used to search the Genecards database (https://www.genecards.org/), the Online Mendelian Inheritance in Man (OMIM) database (https://omim.org/) and the Therapeutic Target (TDD) Database (http://db.idrblab.net/ttd/). The resulting gene targets associated with ALI were collected. Duplicate targets were carefully removed to ensure data accuracy and reliability.

### Protein–protein interaction data

To identify potential therapeutic targets for ALI associated with GRg1, we performed a gene target intersection analysis through Venny 2.1 (https://bioinfogp.cnb.csic.es/tools/venny/index.html)^28^. The common targets obtained from this analysis were further analyzed for their protein–protein interactions (PPI) using the String database (https://cn.string-db.org/)^29^, with the species limited to "Homo sapiens". The PPI data were visualized using Cytoscape 3.9.1 to gain insights into the interactions between these targets.

### Enrichment analysis and molecular docking

To explore the signaling pathways involved with the application of GRg1 in addressing ALI, we performed KEGG analysis and GO analysis by Metascape (https://metascape.org/gp/index.html#/main/step1)^30,31^. These analyses aimed to uncover the biological processes and pathways enriched with the common targets identified earlier. For molecular docking studies, AutoDockTools 1.5.6 and Discovery Studio 2019 were applied^32,33^. The PDB files of the corresponding small molecular ligand substances can be obtained from the RCSB PDB PROTEIN DATA BANK (https://www.rcsb.org/) for performing molecular docking experiments^33^. Additionally, the mol2 Format File for GRg1 was downloaded from the Traditional Chinese Medicine Systems Pharmacology Database and Analysis Platform (TCMSP, https://old.tcmsp-e.com/tcmsp.php) to facilitate the molecular docking analysis^34^.

### Component–disease-target–pathway interaction network construction

To further clarify the potential relationship among components, disease, targets, and pathways, the selected core targets (AKT1, EGFR, VEGFA, SRC, IGF1, ESR1, ALB, and STAT3) and the top 20 enriched KEGG pathways were inputted into Cytoscape 3.9.1 software to construct a component—disease-target—pathway interaction network.

### Molecular dynamics simulations

The molecular dynamics simulations were executed utilizing the non-commercial edition of Desmond/Maestro software, version 2022.1, for molecular modeling and simulations^35,36^. The systems were hydrated with the TIP3P water model and ionic balance was achieved with 0.15 M NaCl. The systems underwent minimization and equilibration, followed by a 100 ns production run in the NPT ensemble, maintaining a temperature of 300 K and pressure at 1 bar. Snapshots of the trajectory were recorded at intervals of 100 ps. Analyses of the molecular dynamics were carried out using the Simulation Interaction Diagram feature available in Desmond.

### Cell culture

MLE-12 cell lines, procured from the American Type Culture Collection (ATCC), were maintained in Dulbecco’s Modified Eagle Medium (DMEM) enriched with 20% fetal bovine serum (FBS) sourced from Gibco (San Diego, California), and incubated at 37 °C within a moisture—saturated environment comprising 5% CO2 and 95% air. Cell subculturing was performed with 0.25% trypsin at a dilution ratio of 1:3. The MLE-12 cells were categorized into four distinct groups for experimental purposes: one control group, two separate groups receiving differing doses of GRg1; and a group serving as the model.

### Quantitative real-time polymerase chain reaction (qRT-PCR)

Genomic RNA was extracted from MLE-12 cells using the SteadyPure Universal RNA Extraction Kit (AG21017; Accurate Biotechnology, Hunan, Co., Ltd.) following the manufacturer's protocol. Subsequently, the extracted RNA was reverse-transcribed into complementary DNA (cDNA) through the Evo M-MLV RT Mix Kit with gDNA Clean for qPCR Ver.2 (AG11728, Accurate Biotechnology, Hunan, Co., Ltd.). We conducted qRT-PCR Utilizing the SYBR Green Premix Pro Taq HS qPCR Kit (ROX Plus) (AG11718, Accurate Biotechnology, Hunan, Co., Ltd.), and the 2-ΔΔCt method was employed for relative gene expression analysis. The primer sequences for qRT-PCR are provided in Table S4.

### Cell Viability analysis

Cell viability was assessed using the CCK8 cell counting kit (New Cell & Molecular Biotech, China). MLE-12 cells were plated at a density of 10,000 cells per well in a 96-well plate and allowed to incubate for 24 h. The absorbance at 450 nm was measured using a microplate reader (Bio-TEK Instrument, USA), and cell viability was calculated using the following formula: cell viability = OD test/OD control, where OD refers to the optical density.

### Western blot

After cell collection, the protein concentrations were assessed utilizing the BCA Protein Assay Kit (Thermo Fisher, Rockford, IL). Individual protein samples were subsequently resolved through SDS‒PAGE electrophoresis and transferred onto PVDF membranes. These membranes were then immersed in TBST buffer containing 5% skim milk for one hour. Primary antibodies, including p-PI3K (#4228), PI3K (#4257), p-AKT1 (#4060), AKT1 (#4691), Bcl-xl (#2764), Bax (#14,796), caspase-3 (#9662), and cleaved caspase-3 (#9661), all diluted at 1:1000, and β-actin (#4970) diluted at 1:5000 and obtained from Cell Signaling Technology, were incubated with the membranes overnight at 4 °C. The membranes were then washed and incubated with the corresponding secondary antibodies for 1 h, and chemiluminescence detection was performed using a contact chemiluminescence instrument (e-BLOT, Shanghai, CN). Protein band quantification was carried out using ImageJ software, and statistical analysis was conducted based on at least three mean values obtained from three independent experiments.

### Apoptosis analysis

MLE-12 cells in the logarithmic growth stage were seeded at a density of 12 × 10^4^ cells per well in 6-well plates. Following the incubation period, MLE-12 cells were collected, washed with PBS buffer, and digested with trypsin. The Annexin V-FITC/PI Apoptosis Detection Kit was used to treat the cells as per the protocol. Subsequently, a flow cytometry analyzer (ACEA, USA) was utilized to analyze apoptosis levels and collect corresponding data.

### Statistical analysis

The results are expressed as the mean ± SEM from a minimum of three independent experiments. Statistical analysis was conducted using one-way ANOVA followed by Bonferroni tests for multiple comparisons with GraphPad Prism software, version 8.0 for Windows (GraphPad Software Inc., USA). For data variables with normal distribution and independent samples, Student’s unpaired t-test was applied to compare the two groups. A significance threshold was set at *p* < 0.05.

### Ethics approval

The animal procedures were conducted following the approval of the Animal Ethics Committee of Guangdong Provincial Hospital of TCM, with ethics number 2020071.

## Conclusions

**Figure 12:**
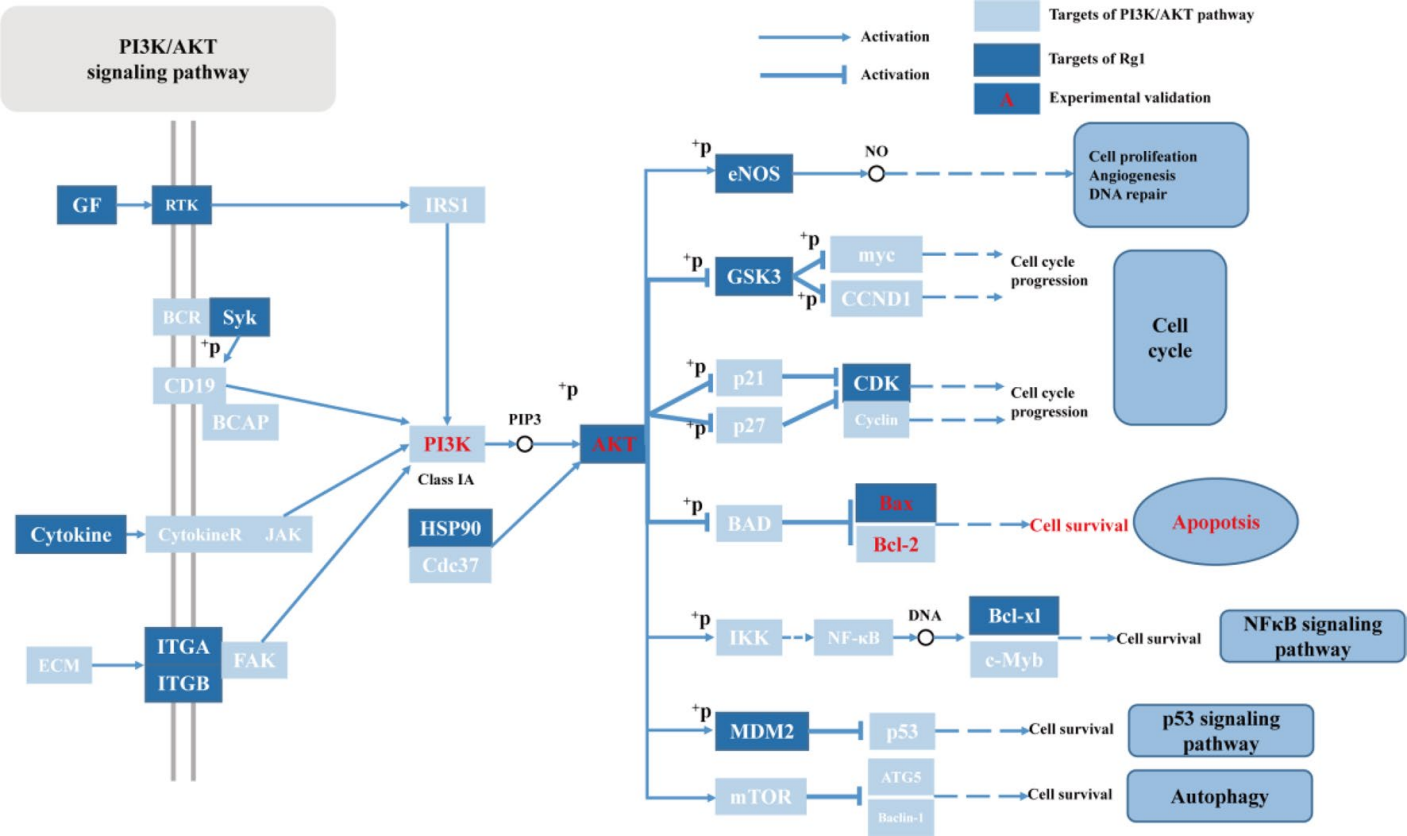
PI3K/Akt-pathway network of the predicted targets of GRg1 enriched in pathways correlated with SALI.

In our research, we employed both experimental models and network pharmacology approaches to forecast and substantiate the therapeutic efficacy of GRg1. The results indicated that GRg1 is an efficacious multi-target therapeutic agent for SALI. Our findings robustly corroborate the protective effect of GRg1 against SALI by reducing apoptosis of AECs via the PI3K-Akt signaling pathway (Fig. 12).

## Supplementary Information

Supplementary Information 1.

Supplementary Information 2.

Supplementary Information 3.

Supplementary Information 4.

## Data Availability

The datasets used and/or analyzed during the current study are available from the corresponding author on reasonable request.
