# Author Correction: The protective effect of ginsenoside Rg1 against sepsis-induced lung injury through PI3K-Akt pathway: insights from molecular dynamics simulation and experimental validation

**DOI:** 10.1038/s41598-024-71998-9

**Published:** 2024-09-11

**Authors:** Kaiqiang Zhong, Yingui Huang, Rui Chen, Qiusha Pan, Jun Li, Xiaotu Xi

**Affiliations:** 1 Second Clinical Medical College, Guangzhou University of Chinese Medicine, Guangzhou, Guangdong China; 2 The Second Affiliated Hospital of Guangzhou University of Chinese Medicine (Guangdong Provincial Hospital of Chinese Medicine), Guangzhou, Guangdong China; 3 Guangdong Provincial Key Laboratory of Research on Emergency in TCM, Guangzhou, Guangdong China; 4 The First Affiliated Hospital of Guangzhou University of Chinese Medicine, Guangzhou, Guangdong China

Correction to: *Scientific Reports* 10.1038/s41598-024-66908-y, published online 11 July 2024

In the original version of this article, the Funding section was incomplete.

“This research was funded by the Natural Science Foundation of China, Grant Number 82204936 and the Basic and Applied Basic Research Project of Guangzhou, Grant Number 2023A04J0476.”

now reads,

“This research was funded by the Natural Science Foundation of China, Grant Number 82204936; the Basic and Applied Basic Research Project of Guangzhou, Grant Number 2023A04J0476; the Guangzhou Municipal Science and Technology Bureau, China (2023A03J0737); and the Guangdong Provincial Key Laboratory of Research on Emergency in TCM (2023B1212060062).”

The original Article has been corrected.

