# Supplementary material for: The protective effect of ginsenoside Rg1 against sepsis-induced lung injury through PI3K-Akt pathway: insights from molecular dynamics simulation and experimental validation
Source: Sci Rep. 2024 Jul 11;14:16071. doi: 10.1038/s41598-024-66908-y (PMC11239675; doi:10.1038/s41598-024-66908-y)
Supplement: Supplementary file 1 — Supplementary Information 1. [file 41598_2024_66908_MOESM1_ESM.pdf]

Figure 1

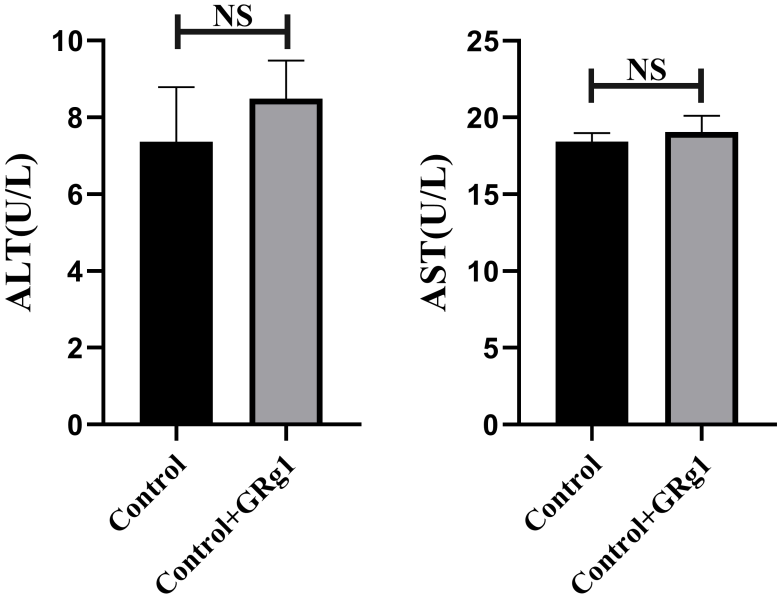
NS
ALT(U/L)
10
8
6
4
2
0
Control
Control+GRg1
NS
AST(U/L)
25
20
15
10
5
0
Control
Control+GRg1

.

Figure 2

广东省中医院实验动物伦理委员会对动物实验研究的审查结果

一、实验项目基本情况：

实验项目名称	人参皂苷 Rg1 对脓毒症树突状细胞免疫功能障碍的干预作用及其机制研究	编号	2020071
动物来源	具有生产资质的供应商	**品种品系**	BALB/c 小鼠 SPF 级
申请单位	广东省中医院	**数量、性别**	530♀
课题负责人	陈瑞	联系人及电话	陈瑞 13120302771
实验目的	探索人参皂苷 Rg1 对脓毒症树突状细胞免疫功能障碍的干预作用及其机制		

二、伦理委员会讨论内容：

参加动物实验研究者资格：	√1、符合条件	2、不符合条件
提供的动物伦理审查材料：	√1、符合条件	2、不符合条件
动物实验的必要性：	√1、必要	2、不必要
是否符合动物福利原则：	√1、是	2、否
动物实验的环境条件是否符合国家标准：	√1、是	2、否

实验方案： √1、合理　　2、基本合理　　3、不合理

三、伦理委员会审议情况：

应到会委员数：5人	实际到会委员数：5人	未到委员及原因：
会议地点： 大学城科学院603会议室		

四、审议结论： 同意实验。

广东省中医院广东省中医药科学院实验动物伦理委员会

委员	签名
曾　星	[signature]
丘小惠	[signature]
郭建文	
郑广娟	
郑起帆	郑起帆
韩凌	
孙景波	[signature]
余谊君	
邓时贵	
郭世宁	[signature]

主任或副主任签名 [signature]
