# Supplementary material for: The protective effect of ginsenoside Rg1 against sepsis-induced lung injury through PI3K-Akt pathway: insights from molecular dynamics simulation and experimental validation
Source: Sci Rep. 2024 Jul 11;14:16071. doi: 10.1038/s41598-024-66908-y (PMC11239675; doi:10.1038/s41598-024-66908-y)
Supplement: Supplementary file 2 — Supplementary Information 2. [file 41598_2024_66908_MOESM2_ESM.pdf]

Table S1: Pharmacological and molecular properties of Ginsenoside Rg1

Name	MW	Hdon	Hacc	RBN	AlogP	TPSA	DL
Ginsenoside- Rg1	801.14	10	14	10	1.13	239.22	0.28

Legend: MW, molecular weight; Hdon, hydrogen bond donors; Hacc, hydrogen bond acceptors; RBN, rotatable Bonds; Alogp, the Ghose-Crippen octanol-water partition coefficient; TpSA, topological polar surface area; DL, drug-likeness.

Supplemental Table S2: The detailed entrez IDs of 116 core targets

NO.	Target	Symbol	Entrez ID
1	Signal transducer and activator of transcription 3	STAT3	6774
2	Interleukin-2	IL2	3558
3	Platelet activating factor receptor	PTAFR	5724
4	Apoptosis regulator Bcl-X	BCL2L1	598
5	Vascular endothelial growth factor A	VEGFA	7422
6	Acidic fibroblast growth factor	FGF1	2246
7	Basic fibroblast growth factor	FGF2	2247
8	Heparanase	HPSE	10855
9	Sodium/potassium-transporting ATPase alpha-1 chain	ATP1A1	476
10	Heat shock protein HSP 90-alpha	HSP90AA1	3320
11	Thymidylate synthase (by homology)	TYMS	7298
12	Integrin alpha-V/beta-3	ITGAV	3685
13	Integrin alpha-IIb/beta-3	ITGA2B	3674
14	Renin	REN	5972
15	Neurokinin 2 receptor	TACR2	6865
16	Serine/threonine-protein kinase AKT2	AKT2	208
17	Rho-associated protein kinase 1	ROCK1	6093
18	RAC-alpha serine/threonine-protein kinase	AKT1	207
19	Galectin-3	LGALS3	3958
20	Integrin beta-3	ITGB3	3690
21	Serine/threonine-protein kinase Chk1	CHEK1	1111
22	Bone morphogenetic protein 2	BMP2	650
23	Mitogen-activated protein kinase 10	MAPK10	5602
24	Steryl-sulfatase	STS	412
25	Apolipoprotein A-II	APOA2	336
26	Complement factor B	CFB	629
27	Transthyretin	TTR	7276
28	Serum albumin	ALB	213
29	Amine oxidase [flavin-containing] B	MAOB	4129
30	Cholinesterase	BCHE	590
31	Tyrosine-protein kinase HCK	HCK	3055
32	Peptidyl-prolyl cis-trans isomerase A	PPIA	5478

33	Aldose reductase	AKR1B1	231
34	Peroxisome proliferator-activated receptor gamma	PPARG	5468
35	SPARC	SPARC	6678
36	Purine nucleoside phosphorylase	PNP	4860
37	Prothrombin	F2	2147
38	Epidermal growth factor receptor	EGFR	1956
39	Alcohol dehydrogenase 1B	ADH1B	125
40	Proto-oncogene serine/threonine-protein kinase Pim-1	PIM1	5292
41	Cathepsin D	CTSD	1509
42	Proto-oncogene tyrosine-protein kinase LCK	LCK	3932
43	BAG family molecular chaperone regulator 1	BAG1	573
44	Heat shock cognate 71 kDa protein	HSPA8	3312
45	Vitamin D-binding protein	GC	2638
46	Cell division protein kinase 2	CDK2	1017
47	Dihydrofolate reductase	DHFR	1719
48	Interferon-stimulated gene 20 kDa protein	ISG20	3669
49	Nitric oxide synthase, endothelial	NOS3	4846
50	cAMP-specific 3,5-cyclic phosphodiesterase 4B	PDE4B	5142
51	Mitogen-activated protein kinase 8	MAPK8	5599
52	Annexin A5	ANXA5	308
53	Liver carboxylesterase 1	CES1	1066
54	Stromelysin-1	MMP3	4314
55	S-methyl-5-thioadenosine phosphorylase	MTAP	4507
56	Serine/threonine-protein kinase 6	AURKA	6790
57	Glutathione reductase, mitochondrial	GSR	2936
58	cAMP-specific 3,5-cyclic phosphodiesterase 4D	PDE4D	5144
59	Complement C1r subcomponent	C1R	715
60	Mitogen-activated protein kinase 14	MAPK14	1432
61	Group 10 secretory phospholipase A2	PLA2G10	8399
62	Oxysterols receptor LXR-beta	NR1H2	7376
63	Proto-oncogene tyrosine-protein kinase Src	SRC	6714
64	Adenosine kinase	ADK	132
65	Estrogen receptor	ESR1	2099
66	Chitotriosidase-1	CHIT1	1118
67	Kinesin-like protein KIF11	KIF11	3832
68	Superoxide dismutase [Mn], mitochondrial	SOD2	6648
69	Progesterone receptor	PGR	5241
70	Tyrosine-protein phosphatase non-receptor type 1	PTPN1	5770
71	Ephrin type-B receptor 4	EPHB4	2050
72	Sex hormone-binding globulin	SHBG	6462
73	Death-associated protein kinase 1	DAPK1	1612
74	Macrophage migration inhibitory factor	MIF	4282
75	Poly [ADP-ribose] polymerase 1	PARP1	142
76	Angiogenin	ANG	283

77	Mineralocorticoid receptor	NR3C2	4306
78	Epoxide hydrolase 2	EPHX2	2053
79	Estradiol 17-beta-dehydrogenase 1	HSD17B1	3292
80	Glutathione S-transferase P	GSTP1	2950
81	Aldehyde dehydrogenase, mitochondrial	ALDH2	217
82	E3 ubiquitin-protein ligase Mdm2	MDM2	4193
83	Alcohol dehydrogenase 1C	ADH1C	126
84	Tyrosine-protein kinase SYK	SYK	6850
85	Phospholipase A2, membrane associated	PLA2G2A	5320
86	Cathepsin B	CTSB	1508
87	Coagulation factor VII	F7	2155
88	Phenylalanine-4-hydroxylase	PAH	5053
89	Alcohol dehydrogenase class-3	ADH5	128
90	Ras-related protein Rab-11A	RAB11A	8766
91	Neutrophil collagenase	MMP8	4317
92	Wiskott-Aldrich syndrome protein	WAS	7454
93	Hexokinase-1	HK1	3098
94	Cathepsin G	CTSG	1511
95	Dipeptidyl peptidase 4	DPP4	1803
96	Collagenase 3	MMP13	4322
97	TGF-beta receptor type-1	TGFBR1	7046
98	Macrophage metalloelastase	MMP12	4321
99	Placenta growth factor	PGF	5228
100	Adenosylhomocysteinase	AHCY	191
101	Carbonyl reductase [NADPH] 1	CBR1	873
102	Glycogen synthase kinase-3 beta	GSK3B	2932
103	Tyrosyl-tRNA synthetase, cytoplasmic	YARS1	8565
104	Urokinase-type plasminogen activator	PLAU	5328
105	Insulin-like growth factor IA	IGF1	3479
106	Cytochrome P450 2C9	CYP2C9	1559
107	Cathepsin S	CTSS	1520
108	Nuclear receptor ROR-alpha	RORA	6095
109	72 kDa type IV collagenase	MMP2	4313
110	Alpha-1-antitrypsin	SERPINA1	5265
111	C-1-tetrahydrofolate synthase, cytoplasmic	MTHFD1	4522
112	Bile acid receptor	NR1H4	9971
113	cAMP-dependent protein kinase, alpha-catalytic subunit	PRKACA	5566
114	Dual specificity protein phosphatase 6	DUSP6	1848
115	MAP kinase-activated protein kinase 2	MAPKAPK2	9261
116	Coagulation factor X	F10	2159

Table S3: Ginsenoside Rg1 molecular docking energy scoring results

Targets	PDB code	Binding energy/kcal·mol-1
ALB	7VR0	-2.12
AKT1	6CCY	-3.85
EGFR	4UV7	-0.68
VEGFA	4KZN	-5.05
SRC	1FMK	-3.36
ESR1	1SJ0	-4.37
IGF1	6FF3	-2.99
STAT3	6NJS	-1.38

Table S4: The information about the primers.

Primer Name	Forward (5'–3')	Reverse (3'–5')
ALB	GCC ACC ATT TGA AAG GCC AG	GCC ACC ATT TGA AAG GCC AG
AKT1	CGT GTG GCA GGA TGT GTA TGA GAA G	CAG GCG GCG TGA TGG TGA TC
VEGFA	CCA CGA CAG AAG GAG AGC AGA AG	GGT CTC AAT CGG ACG GCA GTA G
SRC	GAC CCC TTC GGC CTA TTC AA	CCC ATG GTC CTG GCT GAT AG
ESR1	CCT GGC TGG AGA TTC TGA TGA TTG G	TCC ACC ATG CCT TCC ACA CAT TTA C
IGF1	TTC GCC TCA TTA TCC CTG CC	TAG CCT GTG GGC TTG TTG AA
STAT3	CGA TGC CTG TGG GAA GAG TCT C	ATC TGC TGC TTC TCT GTC ACT ACG
ACTB	GAT TAC TGC CCT GGC TCC TAG	GAA AGG GTG TAA AAC GCA GCTC

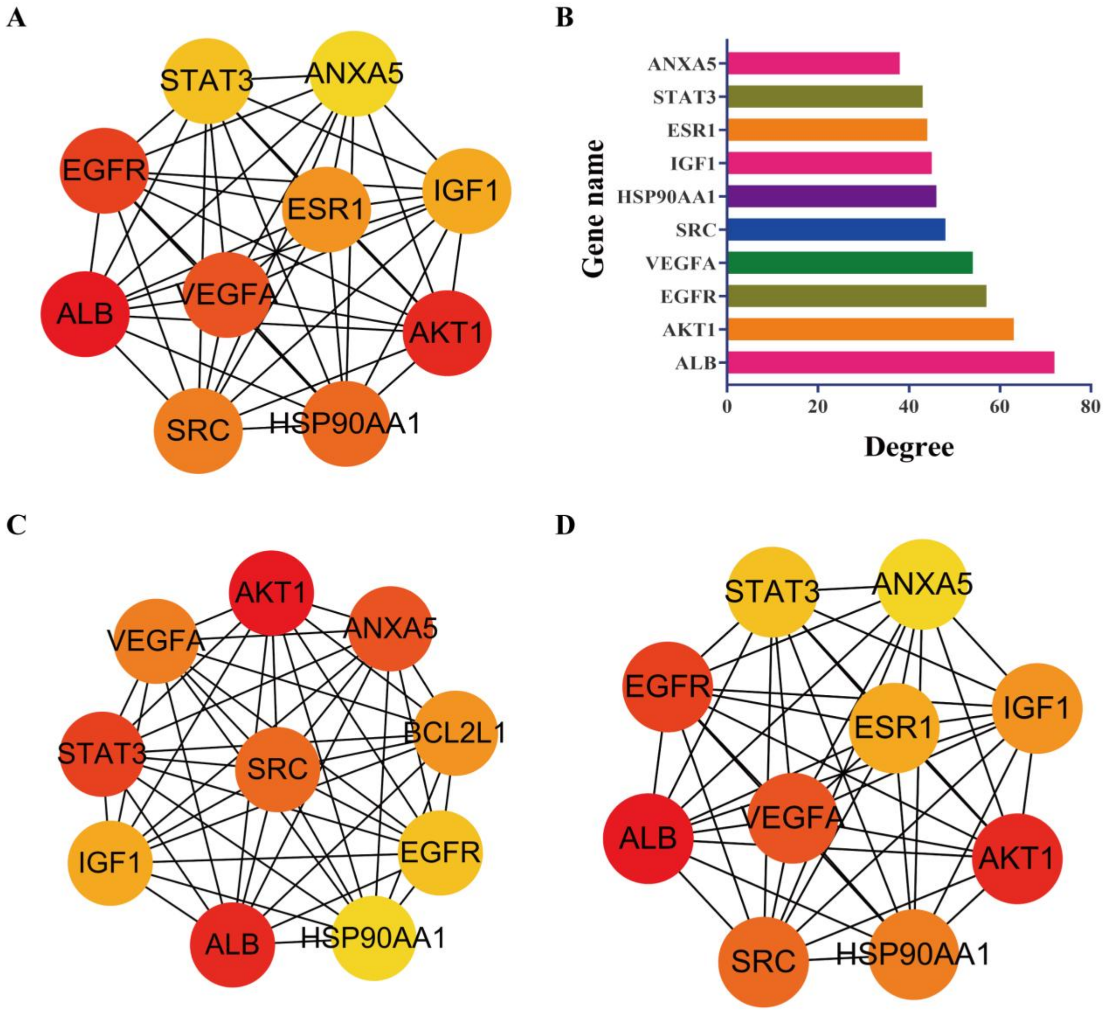

Figure S1. The network of core ALI targets and central ALI targets. The network of top 10 core targets was screened using (A) Closeness, (B) Degree, (C) MCC, and (D) MNC.
