# Supplementary material for: The protective effect of ginsenoside Rg1 against sepsis-induced lung injury through PI3K-Akt pathway: insights from molecular dynamics simulation and experimental validation
Source: Sci Rep. 2024 Jul 11;14:16071. doi: 10.1038/s41598-024-66908-y (PMC11239675; doi:10.1038/s41598-024-66908-y)

Figure 9 Bax

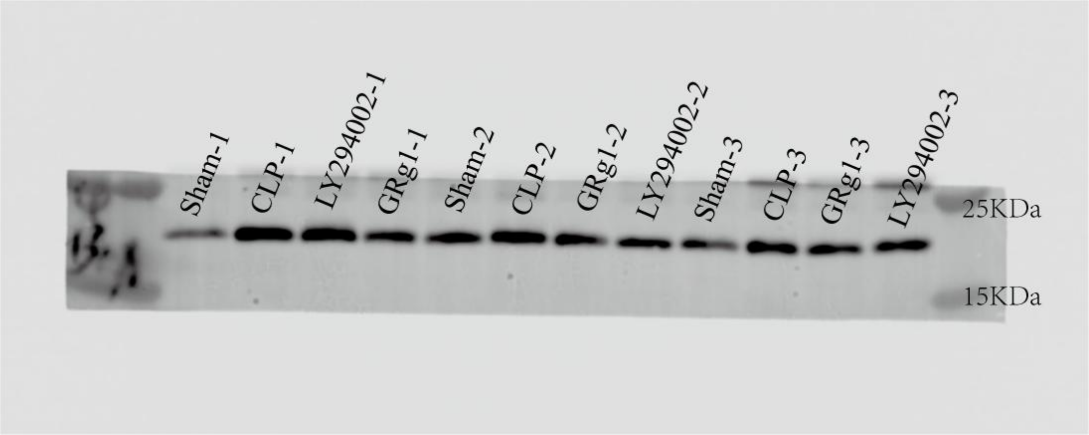

Figure 9 β-actin

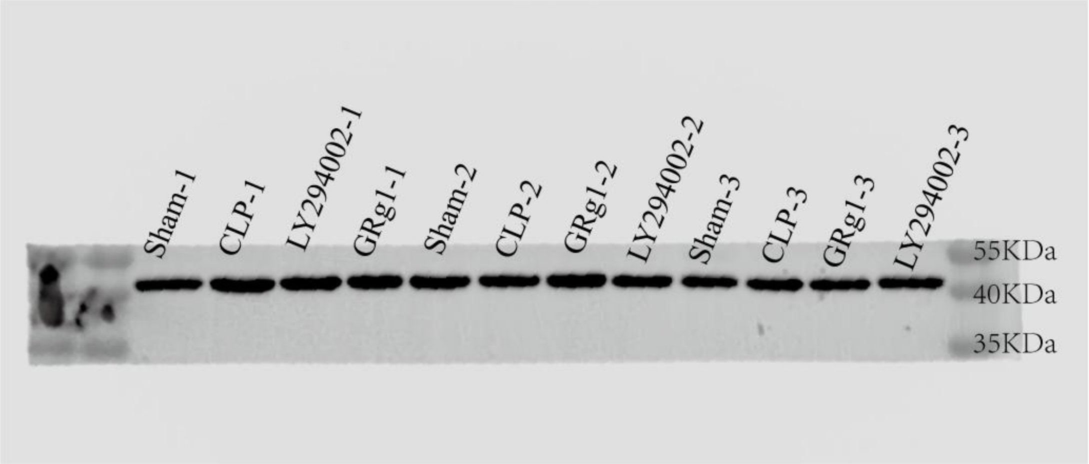

Figure 9 Bcl-xl

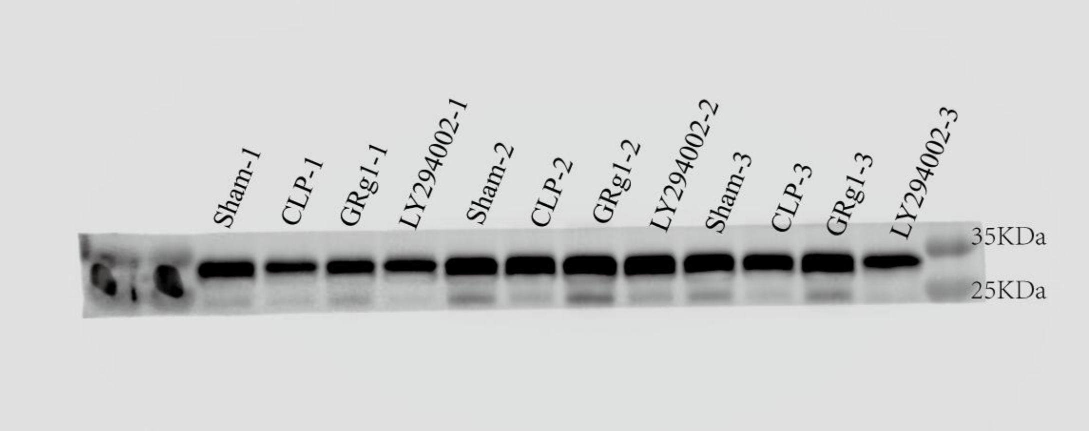

Figure 9 β-actin

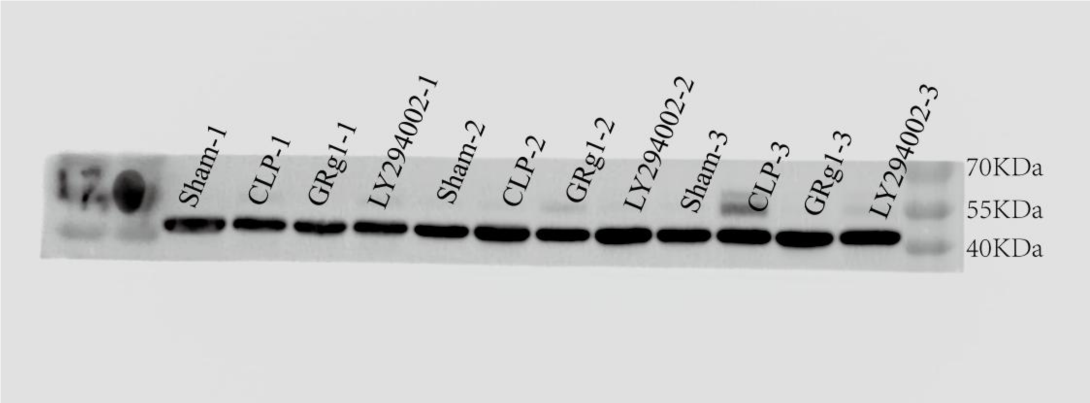

Figure 9 Caspase-3

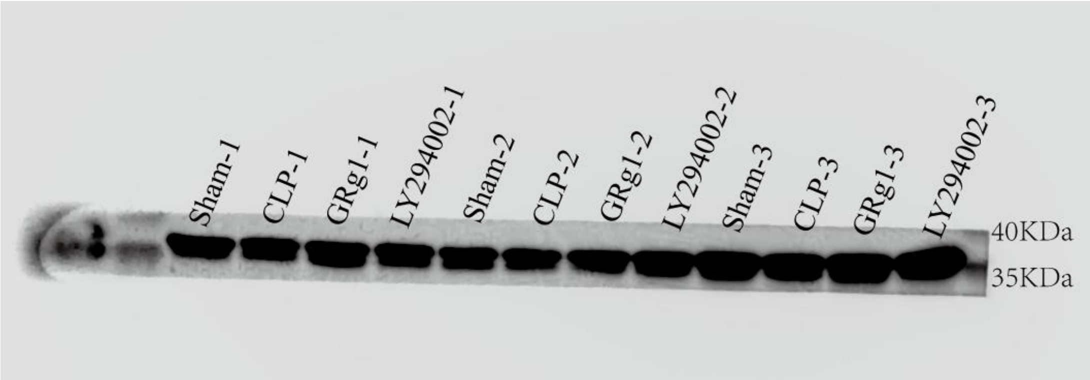

Figure 9 Cleaved caspase-3

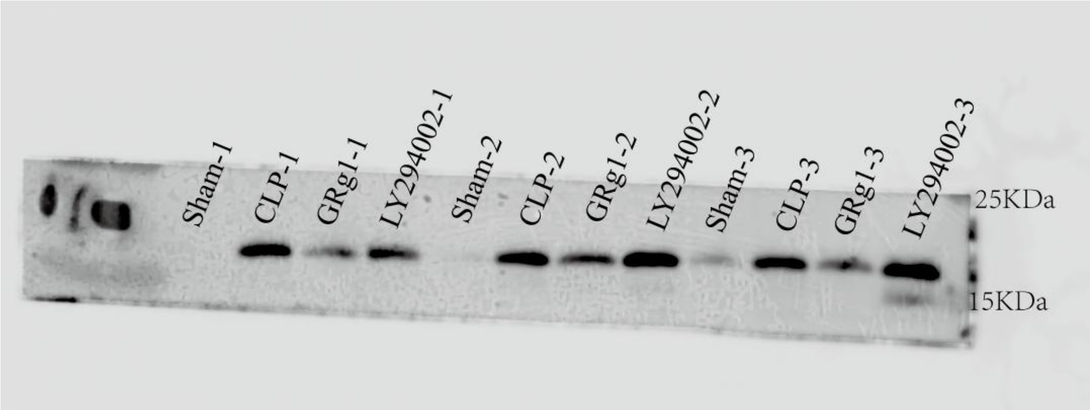

Figure 9 β-actin

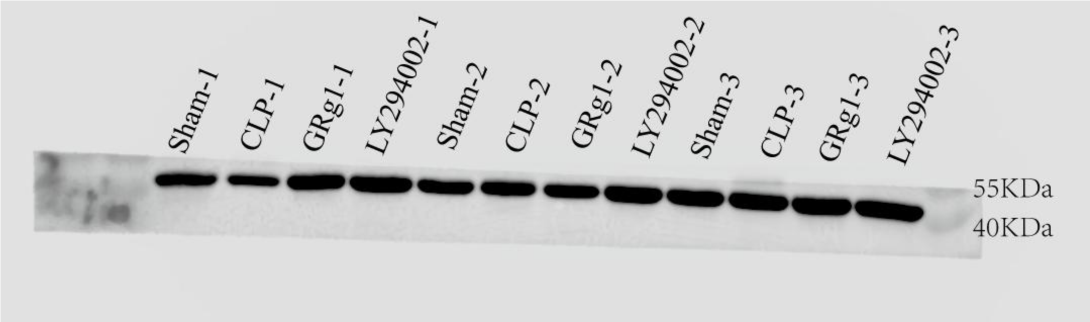

Figure 9 p-AKT

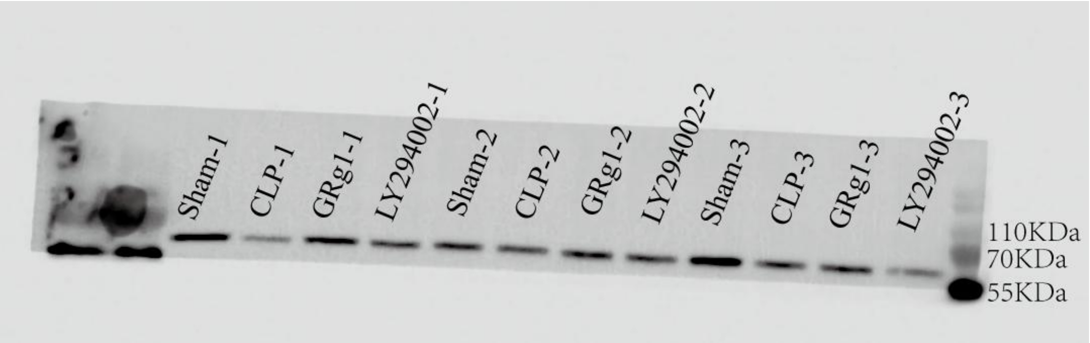

Figure 9 AKT

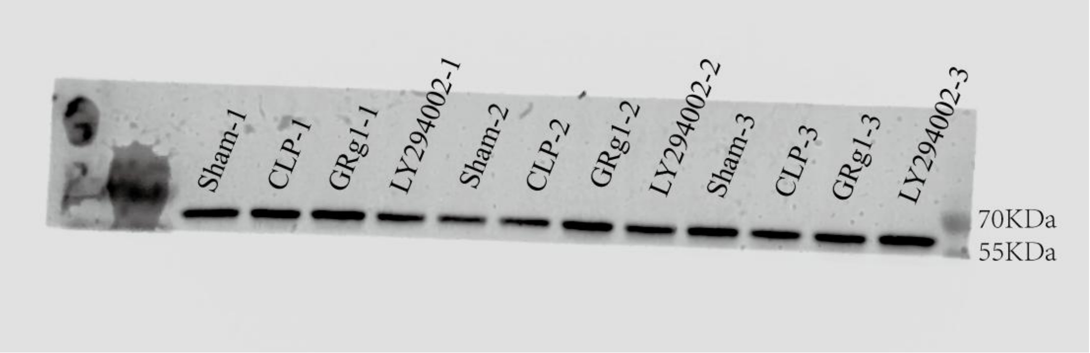

Figure 9 β-actin

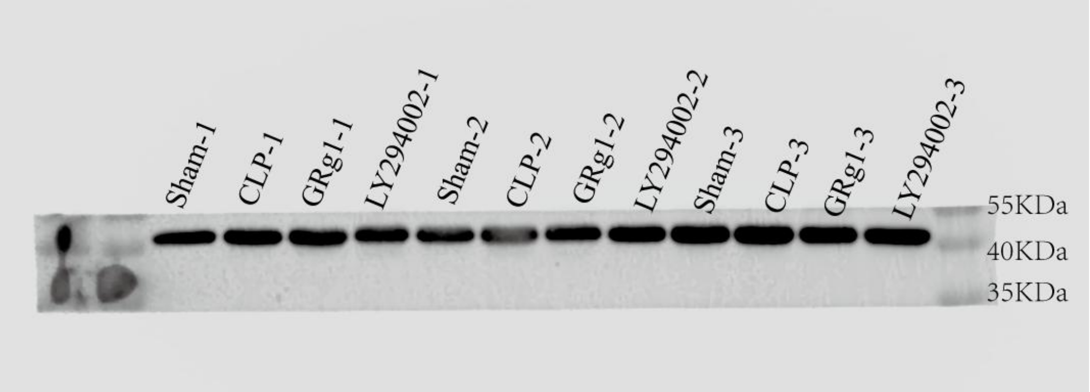

Supplement: Supplementary file 3 — Supplementary Information 3. [file 41598_2024_66908_MOESM3_ESM.pdf]
