# Supplementary material for: The protective effect of ginsenoside Rg1 against sepsis-induced lung injury through PI3K-Akt pathway: insights from molecular dynamics simulation and experimental validation
Source: Sci Rep. 2024 Jul 11;14:16071. doi: 10.1038/s41598-024-66908-y (PMC11239675; doi:10.1038/s41598-024-66908-y)

Figure 7.C

Bax-20kDa

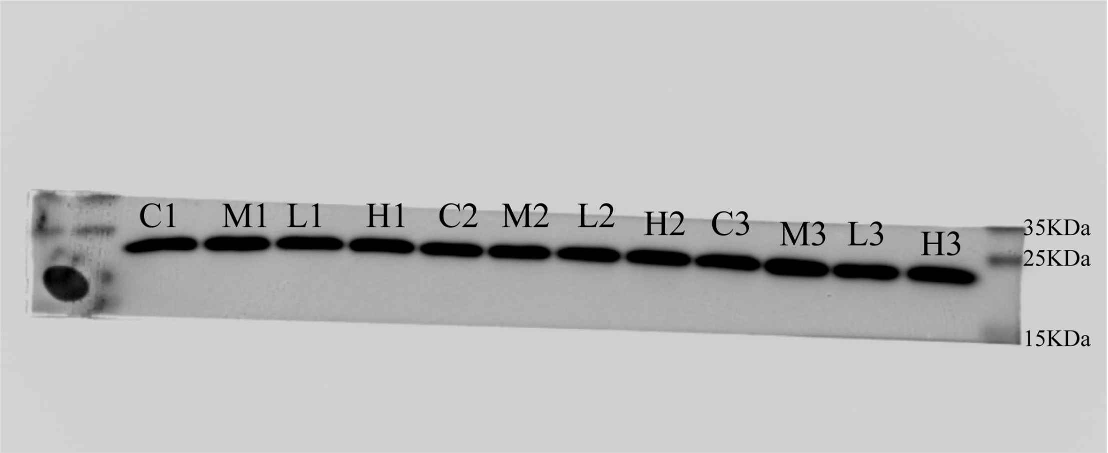

β-actin-42kDa

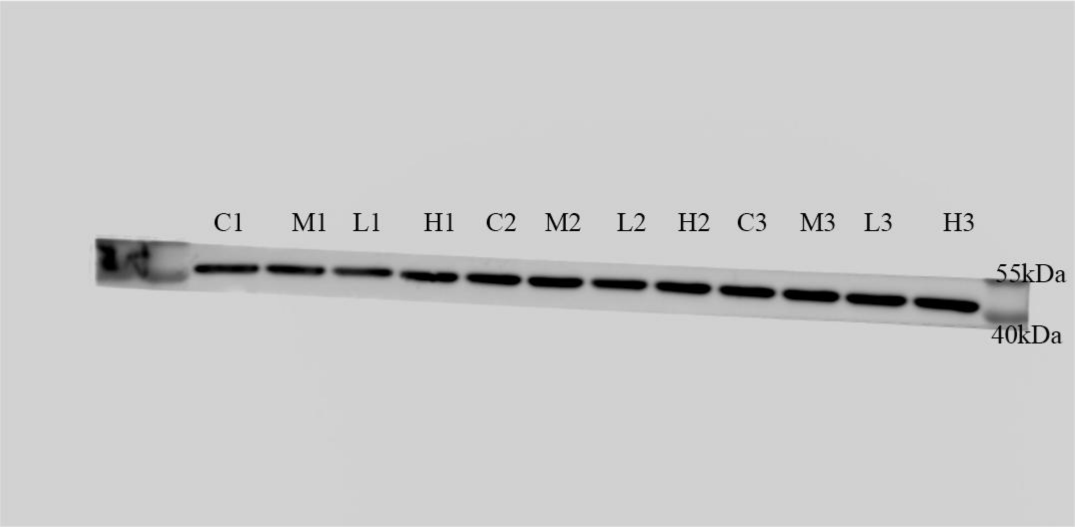

Bcl-xl-30kDa

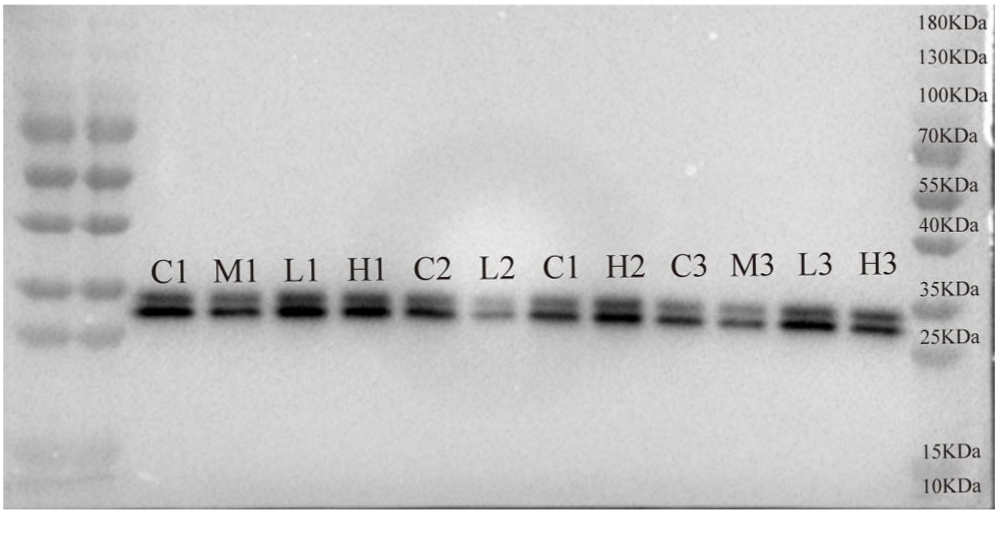

Caspase-3-35kDa

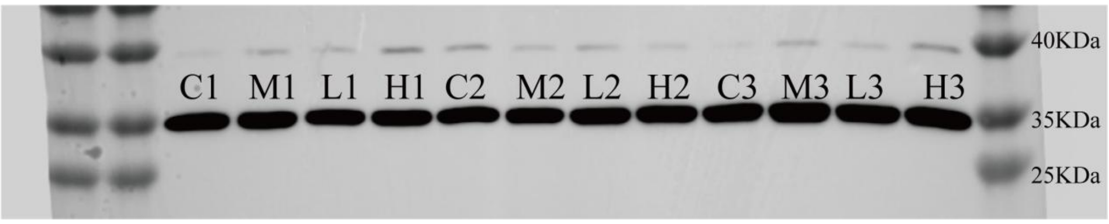

Cleaved caspase 3-17kDa

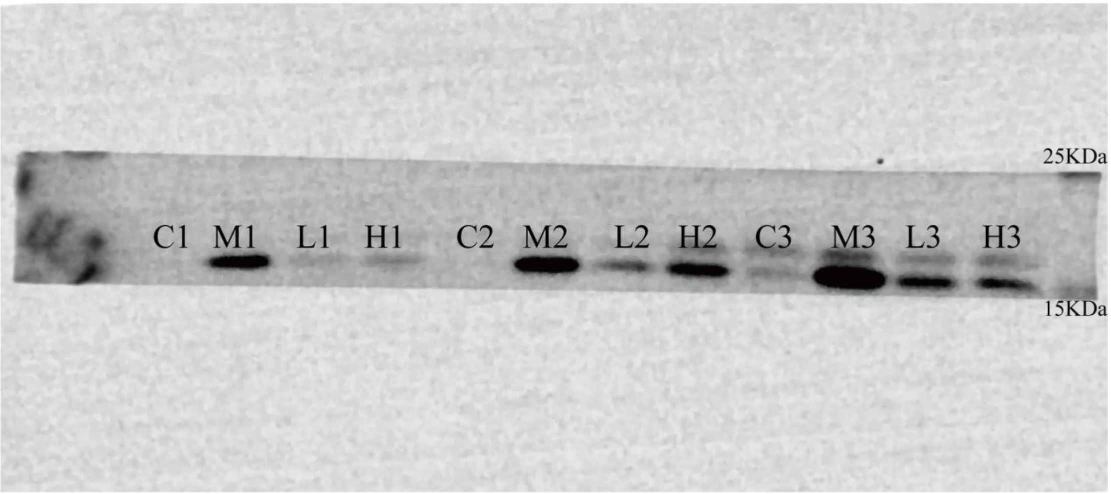

Figure 8.A

P-AKT-60kDa

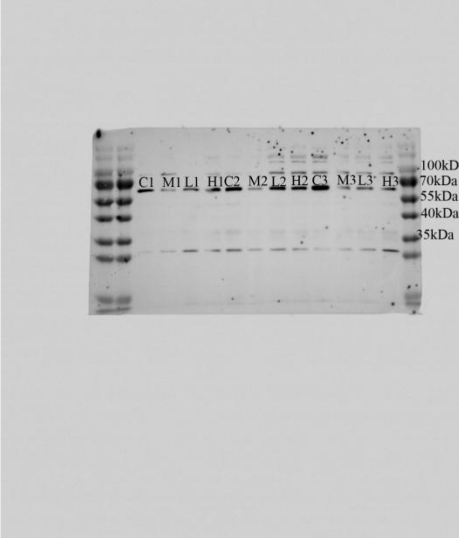

AKT-60kDa

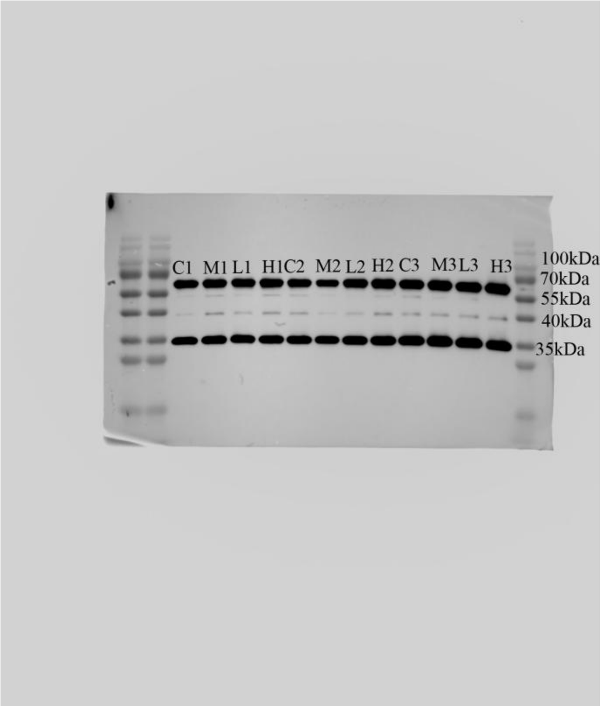

β-actin-42kDa

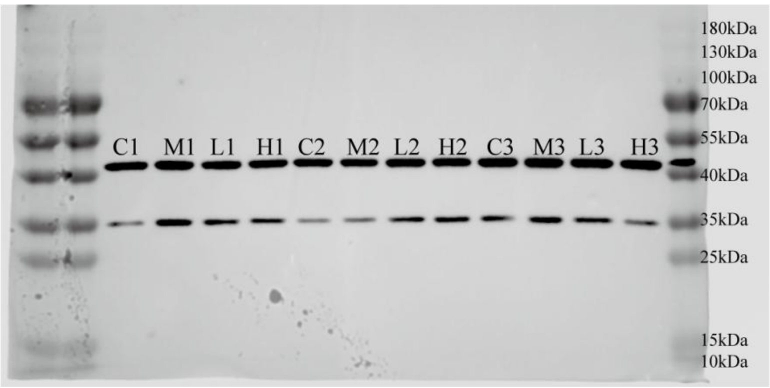

P-PI3K-60kDa

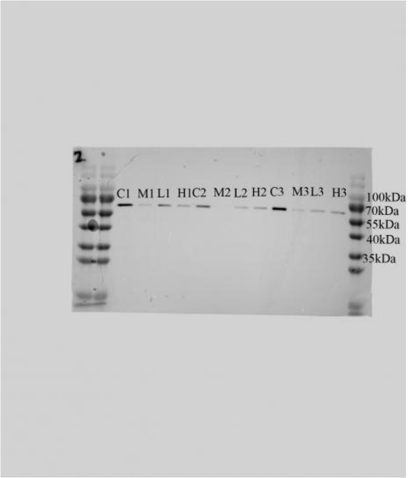

PI3K-85kDa

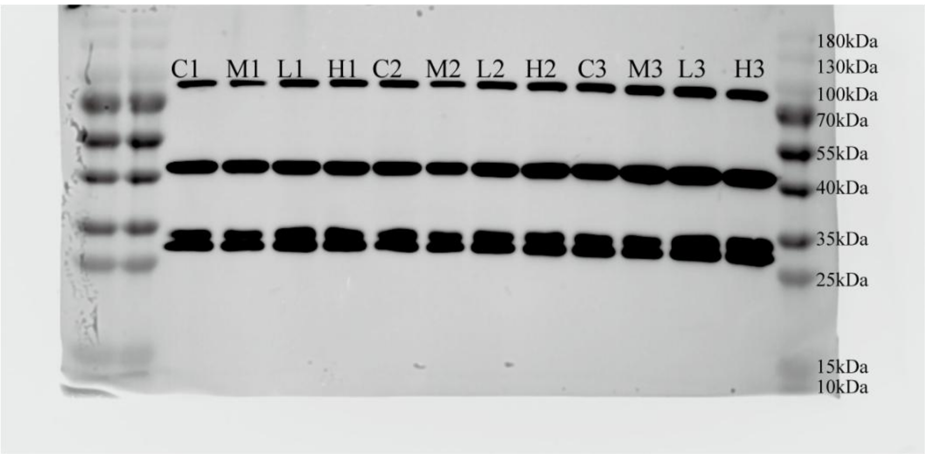

β-actin-42kDa

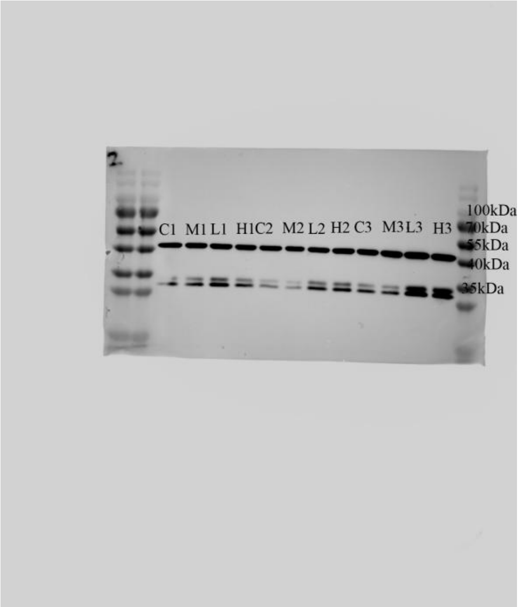

Figure 8.D

p-AKT

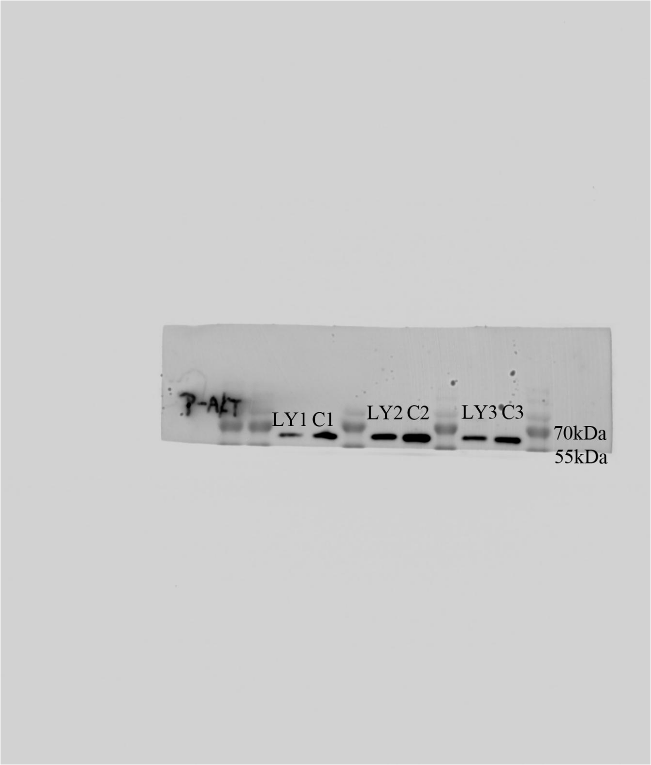

β-actin

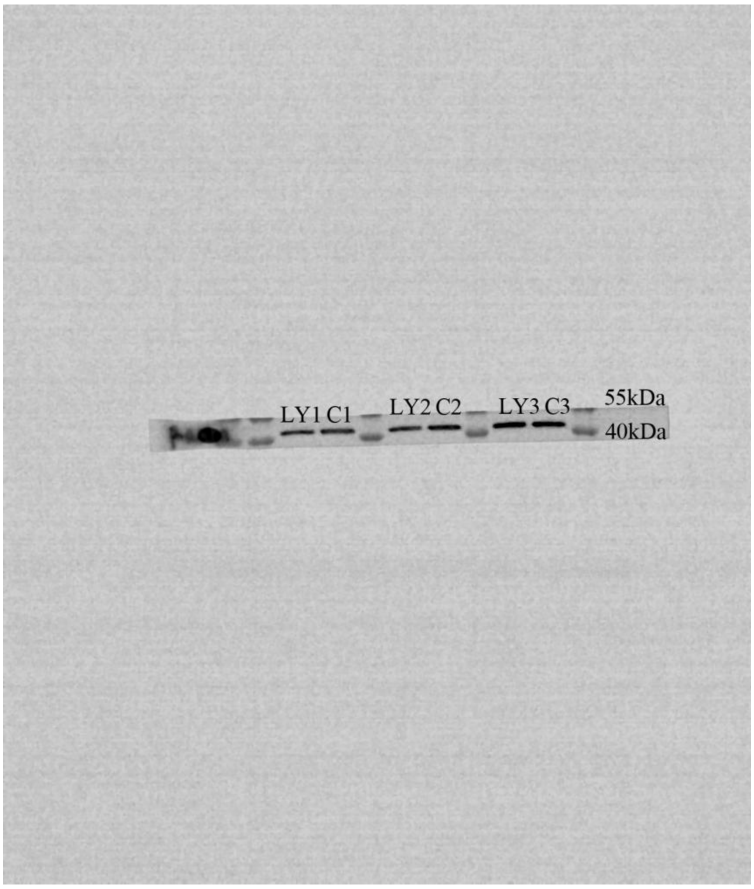

Figure 8.F

p-AKT

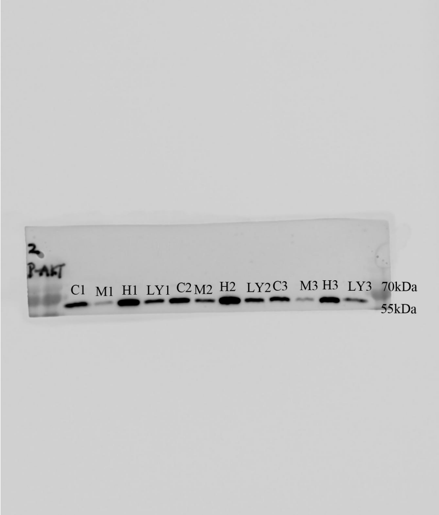

AKT

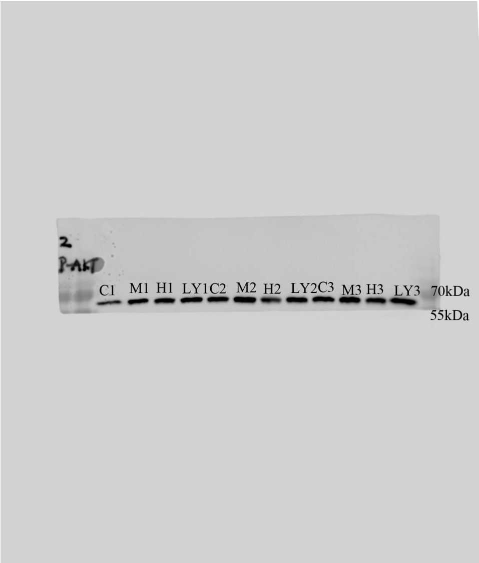

β-actin

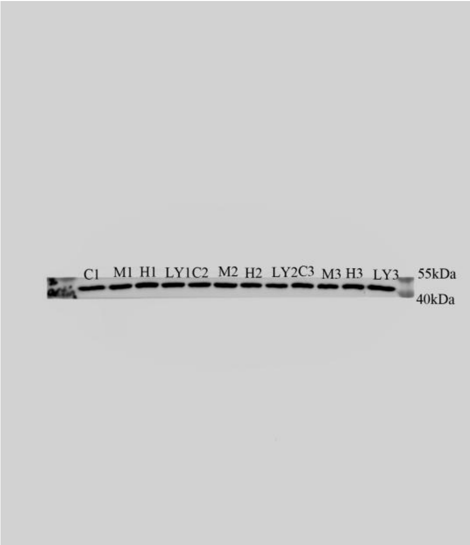

Bax

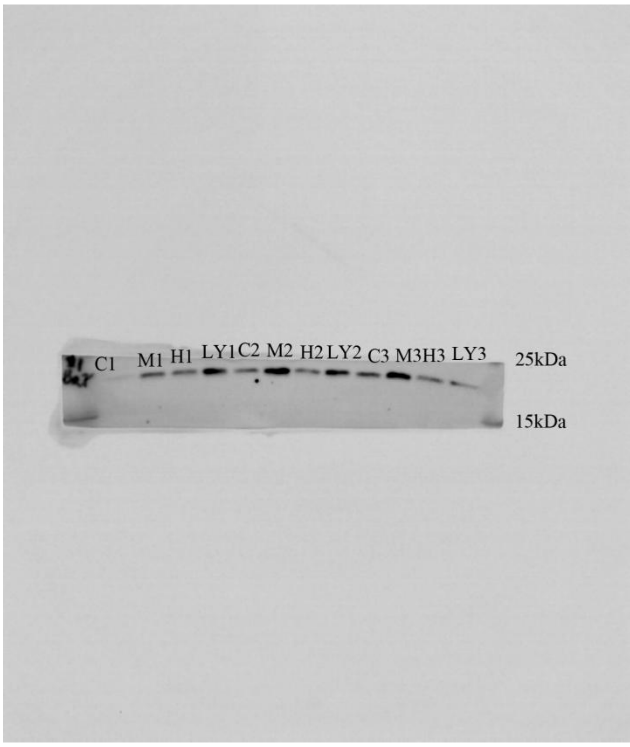
C1
M1
H1
LY1
C2
M2
H2
LY2
C3
M3
H3
LY3
25kDa
15kDa

β-actin

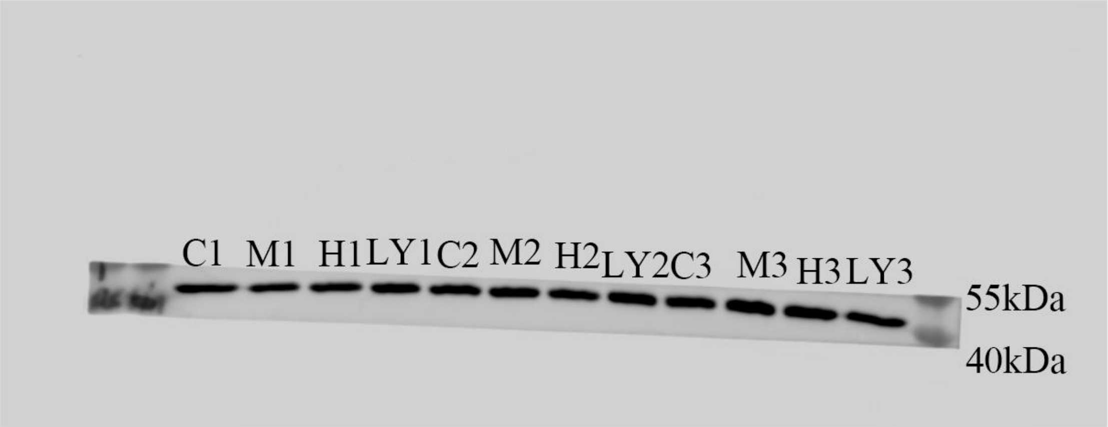
C1
M1
H1
LY1
C2
M2
H2
LY2
C3
M3
H3
LY3
55kDa
40kDa

Figure 8.G
Bcl-xl

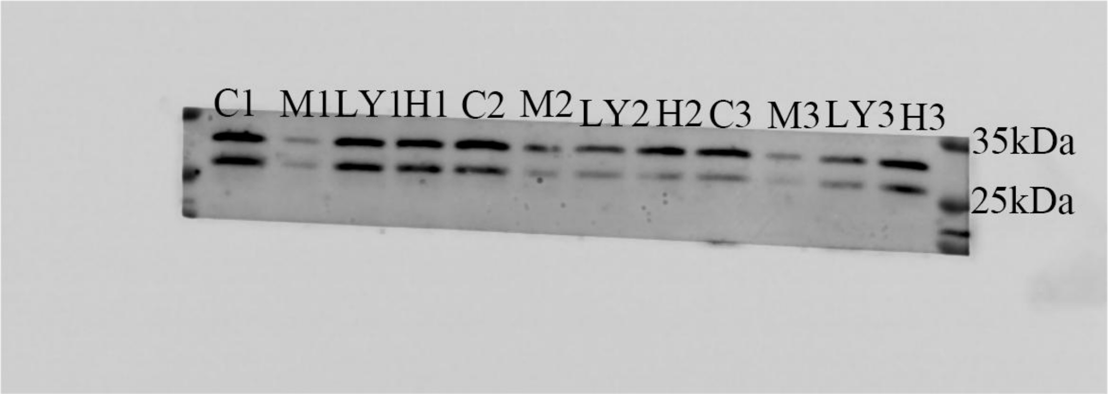

β-actin

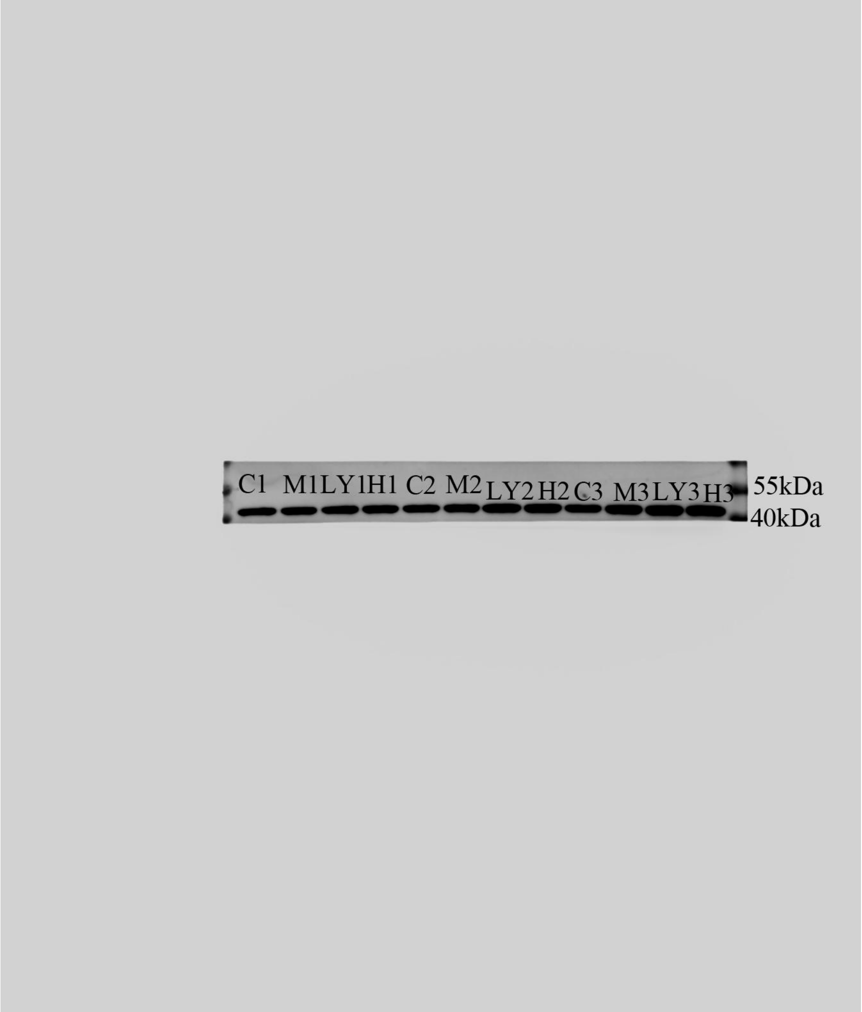

Supplement: Supplementary file 4 — Supplementary Information 4. [file 41598_2024_66908_MOESM4_ESM.pdf]
